# Does combined training of biofeedback and neurofeedback affect smoking status, behavior, and longitudinal brain plasticity?

**DOI:** 10.3389/fnbeh.2023.1096122

**Published:** 2023-01-27

**Authors:** Niki Pandria, Alkinoos Athanasiou, Charis Styliadis, Nikos Terzopoulos, Konstantinos Mitsopoulos, Evangelos Paraskevopoulos, Maria Karagianni, Athanasia Pataka, Chrysoula Kourtidou-Papadeli, Kali Makedou, Stavros Iliadis, Evgenia Lymperaki, Ioannis Nimatoudis, Paraskevi Argyropoulou-Pataka, Panagiotis D. Bamidis

**Affiliations:** ^1^Laboratory of Medical Physics and Digital Innovation, School of Medicine, Faculty of Health Sciences, Aristotle University of Thessaloniki (AUTH), Thessaloniki, Greece; ^2^Department of Psychology, University of Cyprus, Nicosia, Cyprus; ^3^Pulmonary Department-Oncology Unit, “G. Papanikolaou” General Hospital, Aristotle University of Thessaloniki, Thessaloniki, Greece; ^4^Aeromedical Center of Thessaloniki, Thessaloniki, Greece; ^5^Laboratory of Biochemistry, School of Medicine, Aristotle University of Thessaloniki, Thessaloniki, Greece; ^6^Department of Biomedical Sciences, International Hellenic University, Thessaloniki, Greece; ^7^Third Department of Psychiatry, AHEPA University General Hospital, Aristotle University of Thessaloniki, Thessaloniki, Greece; ^8^Aristotle University of Thessaloniki, Thessaloniki, Greece

**Keywords:** smoking cessation, tobacco dependence, stress, electroencephalography (EEG), biofeedback, neurofeedback, EEG-biofeedback, resting-state network

## Abstract

**Introduction:** Investigations of biofeedback (BF) and neurofeedback (NF) training for nicotine addiction have been long documented to lead to positive gains in smoking status, behavior and to changes in brain activity. We aimed to: (a) evaluate a multi-visit combined BF/NF intervention as an alternative smoking cessation approach, (b) validate training-induced feedback learning, and (c) document effects on resting-state functional connectivity networks (rsFCN); considering gender and degree of nicotine dependence in a longitudinal design.

**Methods:** We analyzed clinical, behavioral, and electrophysiological data from 17 smokers who completed five BF and 20 NF sessions and three evaluation stages. Possible neuroplastic effects were explored comparing whole-brain rsFCN by phase-lag index (PLI) for different brain rhythms. PLI connections with significant change across time were investigated according to different resting-state networks (RSNs).

**Results:** Improvements in smoking status were observed as exhaled carbon monoxide levels, Total Oxidative Stress, and Fageström scores decreased while Vitamin E levels increased across time. BF/NF promoted gains in anxiety, self-esteem, and several aspects of cognitive performance. BF learning in temperature enhancement was observed within sessions. NF learning in theta/alpha ratio increase was achieved across baselines and within sessions. PLI network connections significantly changed across time mainly between or within visual, default mode and frontoparietal networks in theta and alpha rhythms, while beta band RSNs mostly changed significantly after BF sessions.

**Discussion:** Combined BF/NF training positively affects the clinical and behavioral status of smokers, displays benefit in smoking harm reduction, plays a neuroprotective role, leads to learning effects and to positive reorganization of RSNs across time.

**Clinical Trial Registration:** https://clinicaltrials.gov/ct2/show/NCT02991781.

## 1 Introduction

Chronic nicotine use has been associated with functional and structural impairment in the central nervous system (CNS; Pomerleau, 1992; Fedota and Stein, 2015; Sutherland et al., 2016) and with progressive deleterious effects to brain function (Musso et al., 2007; Goriounova and Mansvelder, 2012). Smoking-induced atrophy in gray and white matter was reported in multiple widely distributed brain areas but mainly located in frontal, prefrontal, temporal, cingulate, fronto-parietal, and cerebellar sites (Brody et al., 2004; Gallinat et al., 2006; Swan and Lessov-Schlaggar, 2007; Kühn et al., 2012). Moreover, the chronicity of nicotine addiction was positively associated with impairment in periventricular white matter in most of the studies (Fukuda and Kitani, 1996; Kobayashi et al., 1997; Longstreth et al., 2000; Tsushima et al., 2002), while the combination of intensity and chronicity (as in pack-years) was negatively correlated with gray matter density in the prefrontal cortex (PFC; Brody et al., 2004). Resting-state networks (RSNs) including Default-Mode Network (DMN) and others implicated in cognition and executive functions were shown to be affected by addiction (Hong et al., 2009; Weiland et al., 2015). Prefrontal networks involved in attention showed diminished activity to an extent correlated with smoking duration (Musso et al., 2007). In contrast, resting-state connectivity was found to be greater in prefrontal areas which are involved in reward and drug-cue response circuits (Janes et al., 2012). Further, into network function, a disorganization of network topology, correlated with chronicity and severity of smoking dependence was demonstrated, reflected by a decrease of efficiency-related topological properties of DMN and whole-brain networks (Lin et al., 2015). These wide-scale CNS alterations caused by smoking can in part contribute to the explanation of why smoking cessation interventions remain challenging or even largely ineffective (Ashare et al., 2014).

Among non-pharmacological approaches towards facing nicotine addiction and enhancing smoking cessation, biofeedback (BF) and neurofeedback (NF) have been early documented (Griffith and Crossman, 1983; Szalai et al., 1986) to show promising results both clinically and behaviorally. Recently (Pandria et al., 2018), we explored the effects of skin-temperature training on clinical indicators such as exhaled carbon monoxide (CO) and total oxidative stress (TOS) showing a decreasing trend in both indicators. Other studies reported that BF and NF training could decrease tobacco consumption (Griffith and Crossman, 1983; Bu et al., 2019). Several studies have already demonstrated that BF and/or NF could promote gains in behavioral aspects of nicotine addiction such as craving (Canterberry et al., 2013; Hanlon et al., 2013; Li et al., 2013; Kim et al., 2015; Hartwell et al., 2016; Bu et al., 2019), degree of nicotine dependence and presence of psychiatric symptoms (Pandria et al., 2018). NF in particular, as a type of biofeedback that attempts to modulate display of brain activity, is thought to modify brain plasticity naturally, as discussed by Ros et al. (2010). Relevant NF literature as most of the existing real-time functional Magnetic Resonance Imaging (rtfMRI) NF studies revealed a correlation between craving scores and brain activity (Hanlon et al., 2013; Li et al., 2013; Kim et al., 2015; Hartwell et al., 2016). Canterberry et al. (2013), in particular, discussed that low nicotine-dependent individuals may modulate craving-related brain activity more effectively compared to high nicotine-dependent participants, thus suggesting the degree of nicotine dependence may affect training outcomes. Considering that female and male smokers showed differences in smoking behavior (McKee et al., 2005; Lundborg and Andersson, 2008; Buckner and Vinci, 2013; Chinwong et al., 2018), gender could be an important regulatory factor affecting smoking cessation outcomes (Russo and Azevedo, 2010).

The training effects on brain plasticity that could lead to the aforementioned positive behavioral outcomes were investigated by analyzing functional connectivity networks or brain activity. Pandria et al. (2018) explored alterations in the DMN as the latter is found to be affected by smoking (Tanabe et al., 2011; Weiland et al., 2015; Wetherill et al., 2015) and stress (Soares et al., 2013; Clemens et al., 2017), suggesting a positive role of BF-induced stress alleviation to the network affected by nicotine dependence. Bu et al. (2019) explored individualized training of electroencephalography (EEG) patterns using the event-related potential (ERP) component P300 induced by a smoking-cue reactivity paradigm to demonstrate neural plasticity associated with reduced craving. Changes in blood oxygenation level-dependent (BOLD) response were compared between training and baseline scans in most of the rtfMRI studies to evaluate the NF training effectiveness towards modulating craving (Canterberry et al., 2013; Hanlon et al., 2013; Li et al., 2013; Hartwell et al., 2016). Kim et al. (2015) compared functional connectivity between selected brain regions of interest (ROIs) apart from neuronal activity. Other studies assessed targeted components such as skin temperature and specific brain rhythms associated with the training provided (Griffith and Crossman, 1983; Szalai et al., 1986; Grimsley, 1990). For a systematic review of BF and NF investigations on smoking, please see also Pandria et al. (2020). Although BF and NF studies have shown considerable positive changes in the function of the Autonomic Nervous System (ANS; skin temperature, electrodermal activity, heart rate variability; Tolin et al., 2020) and CNS (brain rhythms, brain connectivity; Canterberry et al., 2013; Hanlon et al., 2013; Li et al., 2013; Kim et al., 2015; Hartwell et al., 2016; Pandria et al., 2018; Bu et al., 2019), it remains important to distinguish changes attributed to successful training from non-specific factors such as experience or motivation (Gruzelier, 2014a; Pandria et al., 2020). To this effect, Gruzelier (Gruzelier, 2014a,b,c) extensively discussed theoretical issues that arise while evaluating, validating and reporting neurofeedback studies, highlighting the need for demonstrating feedback learning.

The current manuscript presents and evaluates a multi-visit combined BF and NF training intervention for smoking cessation and focuses on validating learning effects and documenting associated brain plasticity. Building on previous work (Pandria et al., 2018) where we investigated the effects of skin temperature BF training on smoking status, behavior and brain plasticity focusing on the DMN, analysis is hereby extended to include longitudinal clinical, behavioral and electrophysiological data from 17 smokers who completed three evaluation stages of the complete BF/NF intervention protocol of the SmokeFreeBrain project (Pandria et al., 2018; Vardavas et al., 2019). The intervention aimed at combining learning of positive physiological and neurophysiological responses, namely, increased vasodilation induced by skin-temperature BF training (Tolin et al., 2020) and psychological integration induced by alpha-theta protocol NF training (Gruzelier, 2009, 2014a,b,c). Skin-temperature BF training was chosen as an introductory training to operant conditioning. Moreover, skin temperature is considered to be a reliable marker of stress (Karthikeyan et al., 2012) that is involved in different stages of the vicious cycle of smoking addiction (Kassel et al., 2003; Tsourtos et al., 2008). Skin temperature is also affected by nicotine through its sympathetic stimulatory action causing peripheral vasoconstriction (Benowitz and Burbank, 2016). Therefore, skin-temperature BF training seems to be reasonable training for smokers to cope with nicotine effects and stress. Additionally, Hartwell et al. (2013) suggested that the success of BF training can predict the success of NF training. The choice of the alpha-theta protocol was directed by its successful application in other addictions (Peniston and Kulkosky, 1989; Masterpasqua and Healey, 2003; Scott et al., 2005; Sokhadze et al., 2008), post-traumatic stress disorder (Peniston and Kulkosky, 1991), mood improvement (Raymond et al., 2005a) as well as in the creative arts (Egner and Gruzelier, 2003; Raymond et al., 2005b).

We hypothesize that the combination of BF and NF training could improve the smoking status representing the participant’s smoking behavior (Centers for Disease Control and Prevention, 2017). Thus, we aimed to evaluate the training protocol as an alternative smoking cessation approach. Moreover, we assume that training-induced improvement in behavioral measures was expected to be found in anxiety and stress symptoms, depressive symptomatology, self-esteem, quality of life and general health. As such, we aimed to assess the training effect on behavior. Feedback learning was also investigated to validate training effectiveness and capability to promote physiological alterations, distinguishing this from non-specific factors. We considered sex as well as the degree of nicotine dependence as approached by Peng et al. (2018). Greater gains in smoking cessation indices, behavior and training metrics were expected in low/moderate nicotine-dependent smokers. Finally, possible neuroplastic effects of BF/NF training were explored. Whole-brain resting-state functional connectivity networks (rsFCN) were estimated by phase lag index (PLI) for different brain rhythms from EEG recorded at three evaluation stages and were compared across time. The results of rsFCN were interpreted in the context of RSNs as described in Thomas Yeo et al. (2011). Based on existing evidence, our third hypothesis expected neuroplastic changes in RSNs mostly in alpha (8–12 Hz) and theta (4–8 Hz) brain rhythms which were involved in the training (Gruzelier, 2014c). Beta (12–30 Hz) was also examined as it is involved in stress and anxiety (Abhang et al., 2016; Wang et al., 2019).

## 2 Materials and methods

### 2.1 Participants

**Figure 1:**
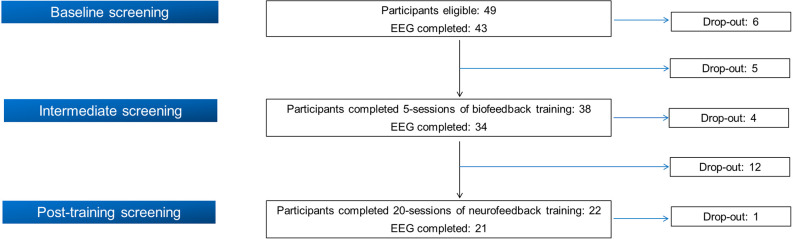
Flow chart of participation in the combined biofeedback/neurofeedback (BF/NF) study.

Initially, 49 smokers were recruited in the BF/NF study following its inclusion and exclusion criteria^1^, implemented in the context of SmokeFreeBrain project. The study protocol was approved by the Ethics and Bioethics Committee of the School of Medicine at the Aristotle University of Thessaloniki (meeting no. 4/2-6-2016 with protocol number: 316/4-7-2016). The work described has been carried out in accordance with the Code of Ethics of the World Medical Association (Declaration of Helsinki). Written informed consent was obtained from all participants. From the initial cohort of 49 participants, 21 completed all training visits as well as the three evaluation stages (see the following section). Figure 1 displays a flow chart of participation and drop-out rate in each stage. Due to the insufficient quality of EEG recordings (body movements, inappropriate electrode placement or malfunction of the EEG apparatus/electrodes) of four participants in different phases, we analyzed complete data from 17 participants (11 females, 64.7%). Their mean age was 48.47 ± 12.05 years, their mean education was 16.76 ± 3.11 years and their mean Body Mass Index (BMI) was 26.35 ± 3.80. Analyzed participants reported a mean nicotine dependence of 27.21 ± 12.18 years while their median pack-years was 18.50 (10.38, 39.50).

### 2.2 Experimental procedures

**Figure 2:**
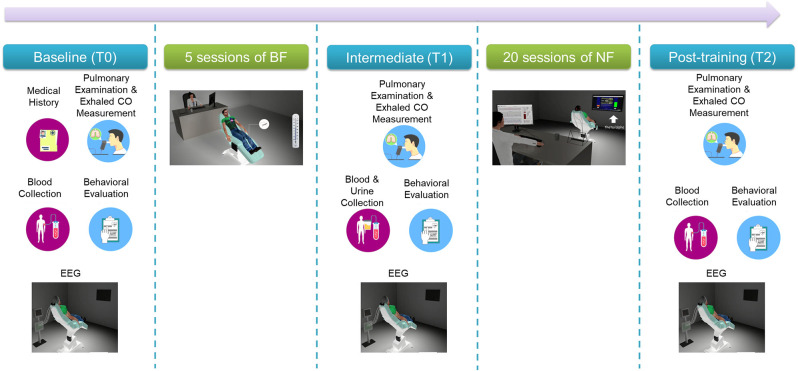
Overview of the experimental procedures.

Participants followed a multi-visit intervention that is shown in Figure 2. Most of the experimental procedures have been previously described in detail (Pandria et al., 2017, 2018).

#### 2.2.1 Baseline screening (T0), intermediate (T1), and final evaluation (T2)

In brief, baseline (T0) screening included two visits: clinical and behavioral examination on visit 1 and an electrophysiological and neuropsychological evaluation on visit 2. Table 1 lists clinical evaluation tools, blood tests, behavioral self-administered questionnaires and neuropsychological tests administered by an experienced neuropsychologist, along with the physiological/psychological function that each item assesses. Following baseline screening procedures, five sessions of BF training were administered in separate visits (Pandria et al., 2018). Upon the completion of BF sessions an intermediate evaluation (T1) was held, with procedures similar to the baseline phase (T0). Subsequently, participants underwent 20 sessions of NF training, in separate visits, following an alpha-theta protocol (Egner et al., 2002; Gruzelier, 2009). Finally, the participants were evaluated (T2) clinically, behaviorally, and electrophysiologically, undergoing the baseline procedures. For each participant who completed the study, the intervention spanned 13 weeks from the 1st visit of baseline evaluation (T0) to the final evaluation visit (T2).

**Table 1:** Overview of clinical, behavioral, and neuropsychological evaluation used in the three stages of the study.

**Test/Questionnaire**	**Administered by/Assesses**
**Clinical**	**pulmonologist**
Spirometry	Pulmonary function estimation
Forced vital capacity (FVC) Forced expiratory flow in 1 s (FEV1) Expiratory ratio (%FEV1/FVC)	
Exhaled carbon monoxide (CO)	Estimation of smoking abstinence
Blood collection	Biochemical markers of smoking
Total oxidative stress (TOS) Vitamin E	
Behavioral	self
Fagerström Test for Nicotine Dependence	Nicotine Dependence
Motivation	Motivation
The contemplation ladder	Readiness to quit smoking
Minnesota Nicotine Withdrawal Scale	Presence of withdrawal symptoms
Beck Depression Inventory	Levels of depressive symptoms
General Health Test	Levels of depressive symptoms
State-Trait Anxiety Inventory	Anxiety symptoms
Rosenberg Self-Esteem Scale	Level of Self-esteem
WHO Quality Of Life Instrument—Brief	Quality of Life
Neuropsychological	neuropsychologist
Stroop Test	Ability to inhibit cognitive interference
Trail Making Test A and B	Visual attention and task switching
Digit Span Test	Working (verbal) memory

#### 2.2.2 Electrophysiological evaluation (EEG)

The electrophysiological evaluation consisted of EEG recording in four different conditions (see Pandria et al., 2018). Resting-state analysis was performed in EEG data during a resting-state with eyes-closed (EC). This EEG recording condition was selected for analysis as resting-state is a task-free condition in which subjects rest quietly awake with EC (Raichle et al., 2001). EEG measurements were carried out in a sound-attenuated and electromagnetically shielded room hosted in Medical Physics Laboratory. A 128-channel EEG recording system (Nihon-Kohden, Japan) was used along with an actiCAP (Brain Products, Germany) with 128 active electrodes placed according to the 10–5 international electrode system (Oostenveld and Praamstra, 2001). The sampling rate was set at 1,000 Hz and electrode impedances were maintained below 10 kΩ.

#### 2.2.3 Biofeedback sessions

Each of the five BF training sessions consisted of skin temperature enhancement through audiovisual feedback. Participants were comfortably seated on a reclined chair in front of a monitor. A commercially available Nexus-10 MKII system (Mind Media, the Netherlands) was used for training along with the Biotrace+ software (V2017A) for Nexus-10. Temperature deviations in their peripheral extremities were measured in Celsius degrees through a Temperature Sensor (Mind Media) designed to detect slight changes (1/1,000th of a degree). A temperature Sensor was placed to the middle fingertip of the non-dominant hand while participants’ fingers were resting on a pillow. Room temperature and humidity levels were also monitored using a digital thermometer (TFA Dostmann/Wertheim, Germany) to ensure that environmental conditions remained stable across sessions. The audiovisual feedback consisted of a self-solving puzzle displayed at the monitor and a pleasant auditory stimulus (Pandria et al., 2016). The feedback was provided when the trainees’ temperature exceeded an auto-adjusted threshold. At the beginning of each training session, stress coping techniques were discussed (15 min) and the peripheral temperature was measured (1 min). Subsequently, 30-min skin temperature training was applied based on suggestions by Peniston and Kulkosky (1989).

#### 2.2.4 Neurofeedback sessions

In each of the 20 NF sessions the participants were lying on a comfortable reclined chair in front of a monitor. An initial orientation regarding the purpose of the applied protocol and its previous applications (Peniston and Kulkosky, 1989; Egner et al., 2002; Raymond et al., 2005a; Gruzelier, 2009; Marzbani et al., 2016), along with instructions, were provided to all participants at the start of the first session. At subsequent sessions, training objectives were reminded. More precisely, the participants were instructed to close their eyes and relax as much as possible, but avoid falling asleep, to breathe slowly and promote positive and relaxing thoughts. At each session, a Minicap manufactured by Mind Media was used to place the active electrode at Pz, ground and reference electrode at right and left earlobe respectively. The training was performed using the same equipment as in BF training (Nexus 10-MKII and Biotrace+ software). EEG was recorded with an ExG sensor (Mind Media). Baseline EEG from the Pz electrode was recorded at rest with EO (2 min) and EC (2 min) both before and after NF training (pre/post). The duration of alpha-theta training was about 40 min per session and sessions were held twice per week. The training goal was to elevate the amplitude of theta rhythm over alpha, facilitating the establishment of a deep relaxation state that resembles a hypnagogic or meditative state (Gruzelier, 2009). Audio feedback was delivered through pleasant sounds (such as a babbling brook or ocean waves crashing on the beach) depending on the production of prominent alpha and theta activity respectively. Two bars were displayed on the monitor that was located in front of participants, one corresponding to the alpha/theta ratio and the other to theta/alpha ratio. The participants’ screen was a pre-defined screen of Biotrace+ software. The methodology of Egner et al. (2002) was followed for feedback delivery that referred to the theta/alpha ratio.

### 2.3 Data processing

#### 2.3.1 EEG analysis

##### 2.3.1.1 EEG preprocessing methodology and source data estimation

Resting-state EEG data recorded during EC condition were pre-processed using Brain Electrical Source Analysis software (BESA research, version 6.0, Megis Software, Heidelberg, Germany). Recordings of each participant were visually inspected for noisy “bad channels”. Ground and reference electrodes (located at AFz, FCz respectively) along with bad channels were interpolated using an incorporated algorithm of BESA software. EEG data were filtered using a low-pass filter of 30 Hz, a high-pass filter of 1 Hz and a notch filter of 50 Hz. Subsequently, we applied an extended version of independent component analysis (ICA; Lee et al., 1999) which includes principal component analysis (PCA) in a current screen of EEG recording (in our case 60 s). PCA is performed before ICA implementation to include PCA components that explain more than 1% of variance. Noisy ICA components were excluded and the EEG dataset was reconstructed. The pre-processing procedure was finalized after the visual inspection of data. Following, 10 random triggers were loaded in the EEG dataset of each participant. Epochs were set in an interval around the triggers (−2,000 ms to +2,000 ms; 4 s in total) and cortical current density reconstruction (CDR) was estimated for all epochs using standardized low-resolution EEG tomography method (sLORETA; Pascual-Marqui, 2002). A generic head finite element model (FEM) was used. Voxel size in Talairach space was set in 10 mm. Four-dimensional CDR images [3-dimensional solution of sLORETA at each time point (4th dimension)] were exported for each subject, each epoch and each evaluation phase (T0, T1, T2). An in-house mask including 863 voxels was applied to these images to eliminate deep sub-cortical areas while keeping cortical and few sub-cortical areas and preserving the sLORETA solution.

##### 2.3.1.2 Functional connectivity analysis

The cortical time-series were extracted from the 4-D images to proceed with functional connectivity (FC) analysis. The connectivity matrix was calculated by applying Phase Lag Index (PLI) using HERMES (Niso et al., 2013). The underlying concept of PLI is to ignore phase differences that are centered around 0 modπ that are mainly attributed to the problem of common sources (Stam et al., 2007) while detecting constant non-zero phase difference that is more likely to indicate a true interaction between two time-series (Nolte et al., 2004; Niso et al., 2013). PLI constitutes an undirected asymmetry measure of the distribution of phase differences (Stam et al., 2007) centered around zero. The distribution of phase differences is considered to be flat if there is no phase coupling between time series. On the other hand, phase synchronization exists if there is any deviation from the flattened distribution of phase differences. The mathematical definition of PLI was introduced by Stam et al. (2007) as follows:


(1) $$PLI=\left|<sig\left[\Delta \varphi \left(t_{k}\right)\right]>\right|$$

PLI values range from 0 that indicates phase coupling around 0 modπ (or no coupling between time-series) to 1 that indicates constant non-zero coupling between time series (which is different from 0 modπ). Although PLI seems to treat the “problem of common sources” reducing Type I errors (Stam et al., 2007), it underestimates phase coupling between time series in the presence of noise (Rubinov and Sporns, 2010) or small-lags between time series resulting in increasing Type II errors (Cohen, 2015).

PLI adjacency matrices were computed for different brain bands (theta: 4–8 Hz, alpha: 8–12 Hz, beta 13–30 Hz) constructing an 863 × 863 × 51 matrix for each brain band (863: number of voxels, 51 = 17 × 3 where 17: number of participants completed the intervention and 3: number of evaluation phases).

Subsequently, two different but complementary approaches were followed. The Network-Based Statistic (NBS) toolbox (Zalesky et al., 2010) was used to compare PLI networks across different evaluation phases (T0, T1, T2) at each brain band. To identify significant connections between PLI networks, the significant level was set at 5%, the number of permutations at 10,000 while the alpha inflation problem due to multiple comparisons was treated through False Discovery Rate (FDR) correction (Zalesky et al., 2010).

Significant networks were visualized using the BrainNet Viewer (Xia et al., 2013). Moreover, the parcellation estimate of the human brain cortex introduced by Thomas Yeo et al. (2011) consisting of seven networks was applied to illustrate the interplay within and between RSNs in the identified significant networks across time. The seven networks are the following: (1) visual network (VIS), (2) somatomotor network (SMN), (3) dorsal attention network (DAN), (4) ventral attention network (VAN), (5) limbic network (LIM), (6) frontoparietal network (FPN), and (7) default mode network (DMN). We applied the same network coloring scheme used by Thomas Yeo et al. (2011).

Additionally, graph properties such as the characteristic path length, mean clustering coefficient, and global efficiency of PLI networks were calculated using the Brain Connectivity toolbox (Rubinov and Sporns, 2010). The aforementioned graph properties were selected due to its implication in nicotine addiction (Lin et al., 2015).

#### 2.3.2 BF and NF training data

Training data were exported from the Biotrace+ software after wiping out artifacts using an automated artifact rejection procedure provided by the software. BF data were tabulated for each participant by averaging the temperature (measured in Celsius) at baseline and during the training. NF data were tabulated for each participant by averaging the amplitude (measured in mV) at pre-training, during the training, and post-training phases. The learning induced by training was explored comparing temperatures (for BF) or amplitude (for NF): (a) across baseline recording of successive sessions, (b) across sessions averages, (c) comparing baseline to the corresponding session average (Gruzelier, 2014c). In the case of NF training, we also, and (d) compared the average amplitude of brain rhythms between pre-training and post-training baseline recordings. Possible alterations within NF sessions will be explored and discussed in a forthcoming manuscript as they are highly dependent on the task or the mental strategy used during separate periods of training (Dempster and Vernon, 2009; Gruzelier, 2014c).

#### 2.3.3 Statistical analysis

Statistical analysis was performed using IBM SPSS (version 26) at a significance level of 5%. Continuous variables were explored for normality assumption using Shapiro-Wilk test (Shapiro and Wilk, 1965), visual inspection of histograms, and boxplots as well as through skewness and kurtosis. Approximately normally distributed variables are denoted as mean±standard deviation while those non-normally distributed as median and interquartile range [IQR, (Q1, Q3) where Q1, Q3 is the first and third quartile of distribution respectively]. Categorical variables are reported as frequencies or percentages.

Quantitative data (clinical, behavioral, learning indices, and resting-state topological parameters) were analyzed within conditions (T0, T1, T2) using either Repeated Measures Analysis of Variance (ANOVA) test or the non-parametric Friedman test depending on test assumptions. In case of non-parametric analysis, Wilcoxon signed-rank test was applied in comparisons across baseline and session data. Alpha inflation problem was handled using Bonferroni correction.

*Post-hoc* tests were performed grouping data with respect to gender and degree of nicotine dependence. Degree of dependence was calculated following the methodology of Peng et al. (2018). Participants were divided into moderate smokers if Fagerström score was below or equal to 6 (11 participants) and severe smokers if Fagerström score was above 6 (six participants). Moreover, participants were divided into two groups depending on pack-years, namely low-dependent and high-dependent smokers. Median pack-years were found to be 18.5. A cut-off of 20 was selected as smoking for more than 20 pack-years is associated with a higher risk of morbidity (Janjigian et al., 2010; McEvoy et al., 2015). None of the participants reported pack-years between 18.5 and 20. Between-group ANOVA analysis could not be implemented as gender and degree of dependence lead to unequal cell sizes that affect hypotheses tested, F-values, and computation of means (Dawn, 1995; Eze and Nwankwo, 2016).

## 3 Results

**Figure 3:**
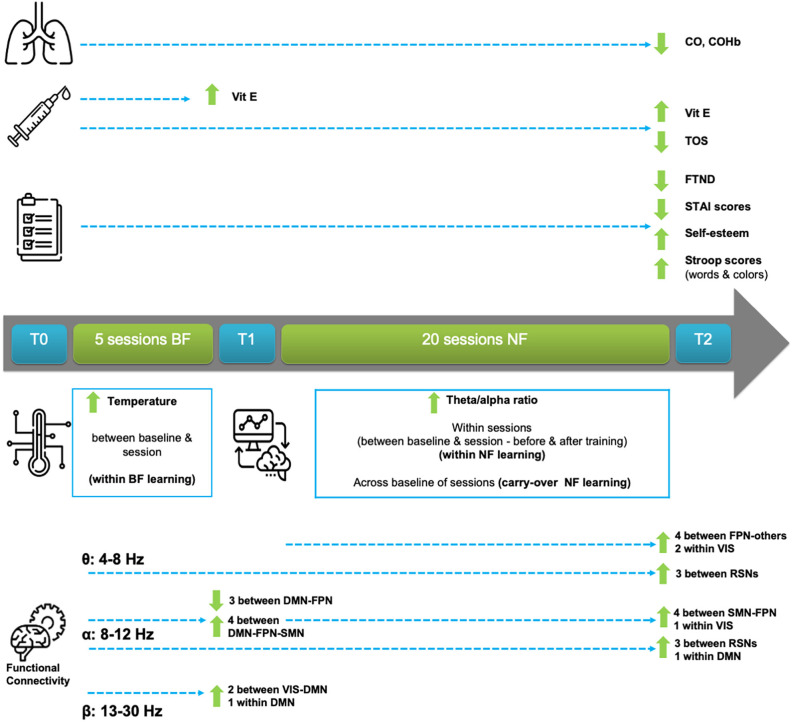
Graphical overview of study’s outcomes.

An overview of the most important clinical, behavioral, feedback training, and electrophysiological outcomes is explained in this Section and is also provided for easier reference in Figure 3. To keep this Section readable, due to the vast amount of results, a detailed statistical analysis report can be found in Supplementary Material S1.

### 3.1 Clinical data

#### 3.1.1 Exhaled carbon monoxide significantly decreased in all participants and low/moderate dependent smokers

Levels of exhaled CO, as well as levels of carboxyhemoglobin (COHb) were measured to be significantly reduced across time (T0, T1, T2). The significance of this finding did not persist when we grouped by gender. Furthermore, we observed a significant decrease in CO and COHb levels in moderate smokers, according to Fagerstrom score, and also in low-dependent smokers, according to pack-years. This decrease was not observed, though, in severe smokers or in high-dependent smokers.

#### 3.1.2 Spirometric parameters remained stable but high-dependent smokers showed decreased forced vital capacity across time

Investigating changes in spirometric measures (FEV1%, FVC%, FEF25%–75%) across time (T0, T1, T2) we did not observe any alteration in all predicted measures for all participants or gender groups.

In grouping by the Fagerström score, we did not find any considerable alteration in all predicted measures for moderate or severe smokers. However, in grouping by pack-years we observed a significant decline in FVC% across time (T0, T1, T2) in high-dependent but not in low-nicotine dependent participants. Pairwise comparisons revealed that FVC% was decreased between T1 and T2 as well as between T0 and T2 in high-nicotine dependent participants.

#### 3.1.3 Considerable improvement was found in both total oxidative stress and Vitamin E levels

Significant changes were found in both total oxidative stress (TOS) and levels of vitamin E across the evaluation phases of the study. Pairwise comparisons between T0 and T2, revealed higher vitamin E levels in T2 compared to T0 in all participants, a finding that was also observed in female participants. Female participants also showed a decreasing trend in TOS and a significant increase in vitamin E levels across all three (T0, T1, T2) evaluation phases. Similar outcomes were observed in male participants.

When grouping by Fagerström scores, TOS was significantly decreased across time (T0, T1, T2) in severe smokers but not in moderate smokers, even though a decreasing trend was observed in the moderate smokers group as well. Vitamin E levels were increased in both moderate and severe smokers across time (T0, T1, T2). Meanwhile, when regarding pack-years grouping, TOS levels decreased only in high-dependent smokers across time (T0, T1, T2). While an increasing trend of vitamin E levels was found in high-dependent smokers, a significant increase was observed only in low-dependent smokers. Pairwise comparisons of vitamin E levels between the three evaluation phases, showed a marginally significant increase between T0 and T2 in both low and high-dependent smokers.

### 3.2 Behavioral data

#### 3.2.1 Self-administered questionnaires reported an improving behavioral profile

##### 3.2.1.1 Decreased nicotine dependence and decreasing trend in withdrawal symptoms

Participants considerably decreased their degree of dependence across time as shown by the Fagerström test of nicotine dependence (Heatherton et al., 1991). Pairwise comparisons indicated that the significant decrease in the degree of nicotine dependence was achieved between T0 and T2. *Post-hoc* tests did not reveal any significant change in the degree of dependence depending on gender. The withdrawal symptoms showed a decreasing trend across time (T0, T1, T2). Grouping by female and male participants, Fagerström score, and pack-years showed similar decreasing trends.

##### 3.2.1.2 Stable motivation, readiness to quit, and quality of life

Motivation and readiness to quit (the Contemplation ladder score) did not show any change across time (T0, T1, T2). *Post-hoc* tests did not reveal changes depending on gender, Fagerström score, and pack-years. Quality of life (QoL) indices were preserved across time in participants. *Post-hoc* tests did not reveal changes depending on gender, Fagerström score, and pack-years for QoL either.

##### 3.2.1.3 Decreasing trends in depressive symptoms and significant decrease in anxiety

Beck Depression Inventory (BDI) scores showed a decreasing trend across time (T0, T1, T2), remaining stable in grouping by gender. However, a decreasing trend was also found in BDI scores across time in grouping by Fagerström score and pack-years.

A significant decrease was found in State Anxiety index (Fountoulakis et al., 2006) across time (T0, T1, T2), showing a decreasing trend also in grouping by gender. Moderate smokers showed a marginal decrease while a decreasing trend appeared in severe smokers. Grouping by pack-years showed that low-dependent participants had decreased State Anxiety scores across time but not the high-dependent group.

Comparing Trait Anxiety scores across time we observed a significant decrease too. Pairwise comparisons showed that the decrease was achieved between T0 and T2. *Post-hoc* tests showed a downward trend across time in both female and male participants, and in moderate smokers but not in severe smokers. The Trait anxiety decrease was found between T0 and T2 in low/moderate dependent smokers. High-dependent participants showed a decreasing trend.

Change in general health test scores was not reported across time in all participants or with any grouping.

##### 3.2.1.4 Increased self-esteem

Self-esteem was found to be enhanced in participants across time (T0, T1, T2). A marginal increase in self-esteem was observed in female participants. Increase in self-esteem across time (T0, T1, T2) and also between T0 and T2 was documented in severe smokers but not in moderate smokers. Grouping by pack-years, both low and high-dependent participants showed an increasing trend.

#### 3.2.2 Neuropsychological assessment

##### 3.2.2.1 Visual attention and task switching increased across time for severe smokers

The completion time of Trail A and B tasks was stable across time in all participants and in grouping by gender. Better performance (decrease in time of completion) in Trail A task was observed in severe/ high-dependent participants across time. Performance did not change in Trail B task for any grouping.

##### 3.2.2.2 Verbal working memory improved in severe smokers but deteriorated in male participants

The participants’ performance at Digit Span Forward task was stable across time in all groupings. Performance at Digit Backward task did not show alterations in all participants but decreased in male participants across time. No changes were found in grouping by Fagerström scores and pack-years. Digit Span total scores did not change for all participants or gender groups across time. Digit Span total scores improved in severe smokers but not in moderate smokers and a marginal increase was revealed in high-dependent but not in low-dependent participants.

##### 3.2.2.3 Cognitive interference inhibition improved in females and moderate smokers

Female smokers improved their Stroop words scores across time (T0, T1, T2) and between T1 and T2, whereas similar findings were not observed in male participants or in other groupings.

Stroop colors task scores did not change. Total Stroop words-colors scores increased across time (T0, T1, T2) and between T0 and T2. Significant improvement in total Stroop words-colors scores was documented in female participants and moderate smokers across time and between T0 and T2. Changes in total Stroop scores across time were revealed in both low-dependent and high-dependent smokers but pairwise significant improvement was shown only in low-dependent subjects between T0 and T2.

### 3.3 Electrophysiological data

#### 3.3.1 Biofeedback

##### 3.3.1.1 Baseline temperature and average temperature across sessions did not increase

Considerable changes in baseline temperature were not observed across sessions for any grouping. Changes in average temperature across sessions were not observed but an increasing trend was documented in sessions 4 and 5 compared to session 1. No changes were observed in post-hoc groupings.

##### 3.3.1.2 Within sessions temperature increased and moderate smokers performed better (baseline vs. session average)

**Figure 4:**
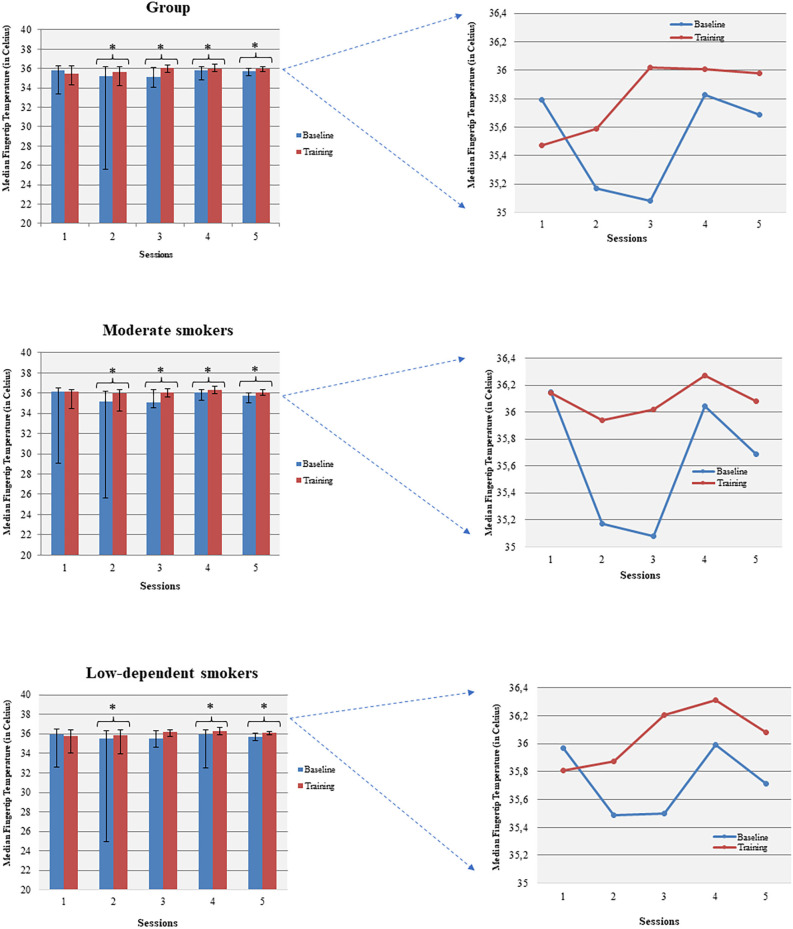
Temperature enhancement was shown across baseline and corresponding sessions (within sessions BF learning). *p<0.05.

Temperature increased within all sessions (apart from session 1) comparing baseline to the corresponding session average temperature (Figure 4). Females increased their average temperature in sessions 4 and 5 while males significantly increased their average temperature in sessions 2, 3, and 5. Moderate smokers increased their temperature during all sessions, apart from session 1, and low-dependent smokers during sessions 2, 4, and 5. Severe/high-dependent smokers increased their temperature during session 3.

#### 3.3.2 Neurofeedback

##### 3.3.2.1 Across sessions all participants and moderate smokers increased baseline theta/alpha ratio but no changes in average amplitudes or ratio was observed

**Figure 5:**
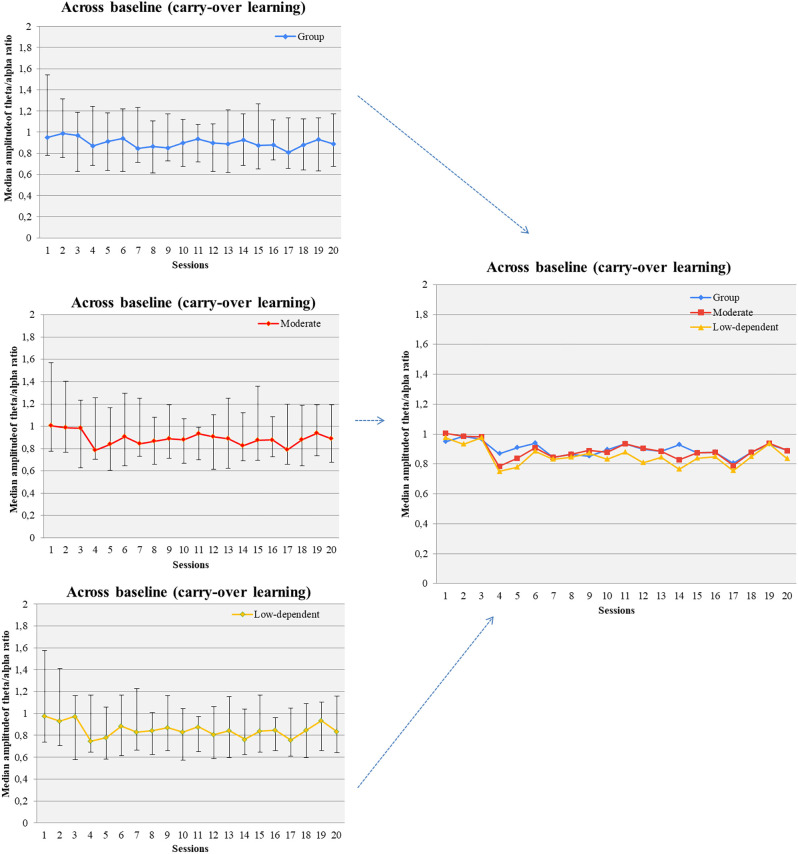
Theta/alpha ratio was increased across baseline measurements of successive NF sessions (carry-over NF learning) in the pool of participants, moderate smokers, and low-nicotine dependent individuals.

Changes in theta and alpha amplitude across baseline of all sessions were not observed. Marginally significant changes were found in the baseline theta/alpha ratio across sessions (Figure 5). Gender grouping showed no significant changes. Low/moderate dependent smokers showed a significant increase of baseline theta/alpha ratio across sessions (Figure 5) but not in baseline measurements in theta and alpha amplitude. On the other hand, average theta amplitude, average alpha amplitude, and average theta/alpha ratio did not change across all sessions for any grouping.

##### 3.3.2.2 Within sessions participants could increase theta amplitude, decrease alpha amplitude, and increase theta/alpha ratio (baseline vs. session average)

**Figure 6:**
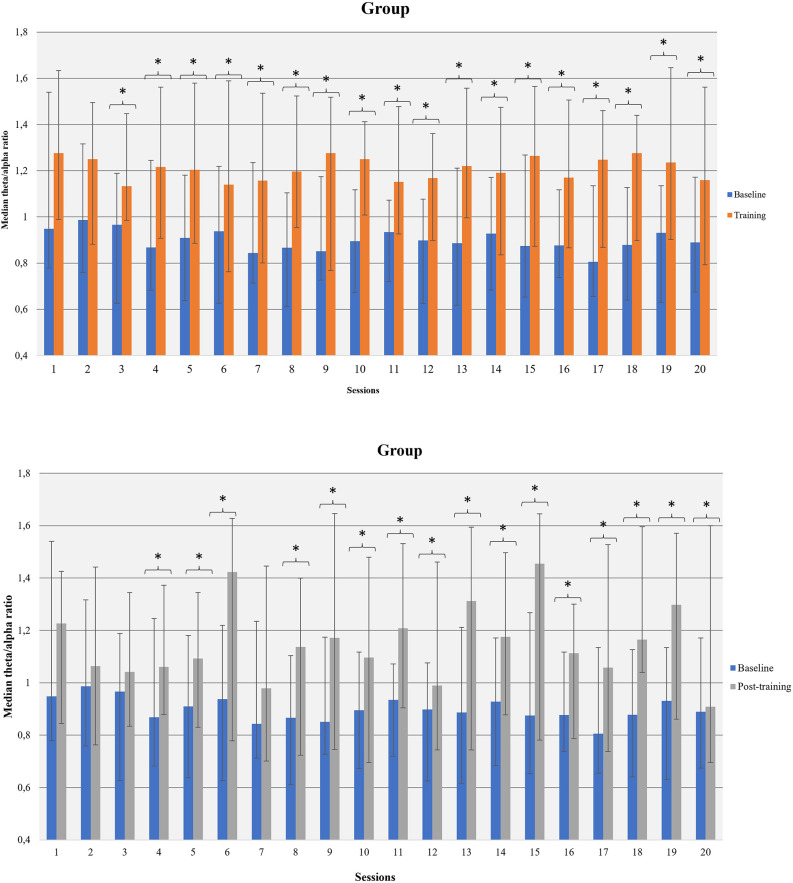
Theta/alpha ratio was increased within NF sessions (between baseline and session average and before-after training; within NF learning) in the whole group of participants. *p<0.05.

Participants increased average theta amplitude within 8th, 15th, and 17th sessions compared to the corresponding baseline measurements. The amplitude of alpha-band decreased in sessions 2, 4, 8, 11, 12, 19, and 20. An increase in alpha amplitude, relative to the corresponding baseline, was observed in sessions 10 and 13. The average theta/alpha ratio was found to be higher during sessions compared to the baseline, in all sessions apart from the first and second (Figure 6).

Female participants increased the average theta/alpha ratio in all sessions apart from session 1 and session 7. Male participants increased theta/alpha ratio in sessions 4, 7, 8, 9, 10, 12. Moderate smokers enhanced their theta/alpha ratio in sixteen (16) out of 20 sessions compared to the corresponding resting-measurement while severe smokers in twelve (12) out of 20 sessions. Low-dependent individuals increased their theta/alpha ratio in fourteen (14) out of 20 sessions while high-dependent smokers in eighteen (18) out of 20 sessions.

##### 3.3.2.3 After training participants had increased theta, decreased alpha and increased theta/alpha ratio compared to the baseline measurements, especially females and moderate smokers (within sessions pre-post)

Participants showed enhanced theta amplitude in 6th, 15th, and 20th post-training measurements compared to the corresponding baseline. Theta amplitude was increased in sessions 6 and 15 for female participants and in sessions 8, 12, and 17 for males. Moderate smokers showed higher theta amplitude after training in 7 out of 20 sessions. We did not observe changes in theta amplitude in severe smokers. Likewise, low dependent smokers showed increase in theta amplitude after training in 5 out of 20 sessions compared to baseline whereas no changes were observed in high-dependent smokers.

Comparing alpha amplitude before and after NF training (within each session), we found a decreasing trend in all sessions apart from 12 to 13. A decrease in alpha amplitude was found in two (2) out of 20 sessions (8th, 9th) for female participants but no changes were observed in male participants. A decrease in alpha amplitude after training was indicated at the 8th session for moderate smokers and the 11th session for severe smokers.

Theta/alpha ratio was increased after training compared to the corresponding baseline in all sessions apart from the first three sessions and 7th session (Figure 6). Female participants had increased theta/alpha ratio after training in twelve (12) out of 20 sessions and males in one (1) out of 20 sessions. Moderate smokers had increased theta/alpha ratio in eleven (11) out of 20 sessions after training whereas severe smokers in two (2) out of 20 sessions. Likewise, low-dependent smokers had increased theta/alpha ratio in eight (8) out of 20 sessions and high-dependent smokers in two (2) out of 20 sessions.

#### 3.3.3 Electroencephalography

##### 3.3.3.1 Theta and alpha phase lag index networks mainly were highly modified across time involving different resting-state networks

**Figure 7:**
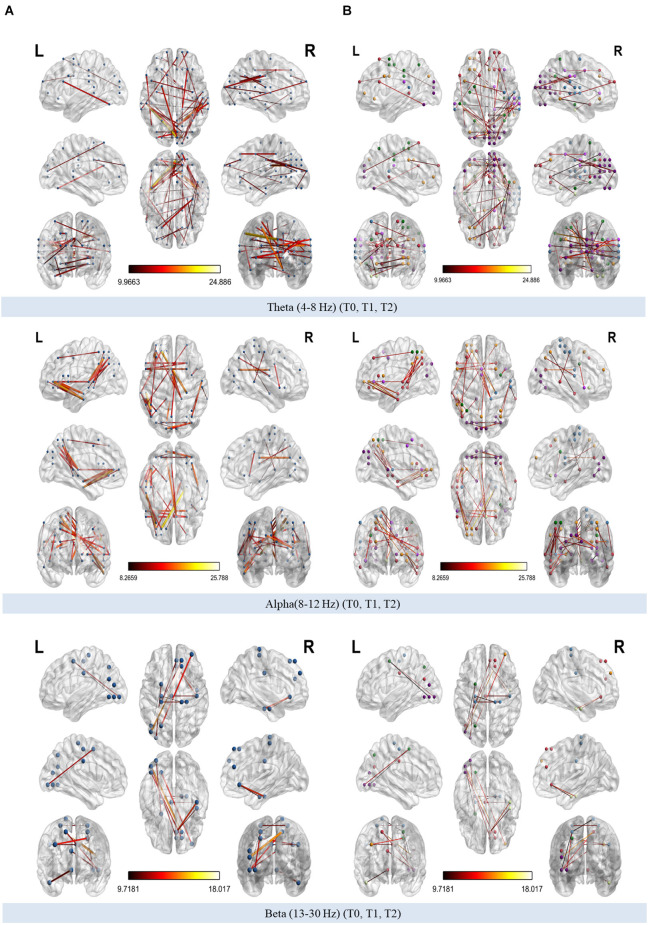
**(A)** Modified connections of phase-lag index (PLI) networks (theta, alpha, and beta bands respectively) across time. **(B)** Modified connections of theta, alpha, and beta PLI networks across time annotated according to 7-network estimate described by Thomas Yeo et al. (2011).

The organization of theta, alpha, and beta PLI networks were found to be modified across time (T0, T1, T2; Figure 7). Coloring network’s nodes based on Thomas Yeo et al. (2011) 7-network estimate, those significant connections that emerged both from the interaction across the three time points as well as from pairwise comparisons between T0-T1, T1-T2, T0-T2 could be considered as either within or between RSN connections. The direction of significant change in synchronization between nodes was also marked as increased or decreased when those connections were also identified in pairwise comparisons (Tables s 2–4).

**Table 2:** Different connections found in theta PLI networks across time (T0, T1, T2) and after pairwise comparisons (L denotes left hemisphere; R denotes right hemisphere).

**Different connections found in theta PLI networks across time (T0, T1, T2)**					**RSNs**
R Inferior Frontal Gyrus	R Sub-Gyral (BA47)				LIM—DAN
R Inferior Frontal Gyrus	R Sub-Gyral (BA47)				DMN—DAN
R Parahippocampal Gyrus	L Inferior Frontal Gyrus				DAN—FPN
R Uncus	L Inferior Frontal Gyrus				LIM—FPN
R Parahippocampal Gyrus	L Inferior Frontal Gyrus				DAN—FPN
R Lingual Gyrus	L Superior Temporal Gyrus				VIS—DMN
R Inferior Frontal Gyrus	L Middle Frontal Gyrus				Within FPN
R Medial Frontal Gyrus	L Middle Frontal Gyrus				DMN—FPN
R Superior Temporal Gyrus	R Cuneus				SMN—VIS
R Superior Temporal Gyrus	L Cuneus				SMN—VIS
R Posterior Cingulate	R Middle Occipital Gyrus				Within VIS
L Middle Occipital Gyrus	R Cuneus				Within VIS
L Superior Temporal Gyrus	R Cuneus				DMN—VIS
L Middle Occipital Gyrus	R Postcentral Gyrus				VIS—SMN
L Cuneus	R Cuneus				Within VIS
R Superior Temporal Gyrus	L Inferior Parietal Lobule				SMN—VAN
R Precuneus	R Inferior Parietal Lobule				VIS—VAN
R Posterior Cingulate	R Inferior Parietal Lobule				DMN—VAN
L Superior Temporal Gyrus	R Superior Frontal Gyrus				SMN—DMN
R Middle Temporal Gyrus	R Superior Frontal Gyrus				FPN—DMN
R Middle Occipital Gyrus	L Precuneus				VIS—FPN
R Inferior Parietal Lobule	R Precuneus				SMN—DMN
R Transverse Temporal Gyrus	R Angular Gyrus				SMN—DAN
R Cuneus	L Postcentral Gyrus				VIS—DAN
R Middle Occipital Gyrus	R Postcentral Gyrus				VIS—VAN
R Precuneus	R Inferior Parietal Lobule				VIS–VAN
R Middle Occipital Gyrus	R Precentral Gyrus				VIS—SMN
R Middle Occipital Gyrus	R Precentral Gyrus				VIS—VAN
L Superior Frontal Gyrus	L Cingulate Gyrus				DMN—FPN
R Middle Frontal Gyrus	R Medial Frontal Gyrus				DMN—FPN
R Anterior Cingulate	R Precuneus				DMN—VIS
R Cuneus	L Postcentral Gyrus				VIS—DAN
R Cuneus	L Postcentral Gyrus				VIS—DAN
R Cuneus	L Postcentral Gyrus				VIS—DAN
R Cuneus	R Cingulate Gyrus				VIS—VAN
R Paracentral Lobule	R Superior Frontal Gyrus				VAN—DMN
R Lingual Gyrus	L Precuneus				VIS—DAN
R Superior Temporal Gyrus	L Precentral Gyrus				SMN—DAN
R Cuneus	L Sub-Gyral (BA47)				VIS—DAN
L Middle Frontal Gyrus	R Precentral Gyrus				FPN—SMN
R Cuneus	L Superior Frontal Gyrus				VIS—DAN
L Cuneus	L Superior Frontal Gyrus				VIS—DMN
Pairwise comparisons: ↑ ↓					RSNs
		T0 vs. T1	T1 vs. T2	T0 vs. T2	
R Parahippocampal Gyrus	L Inferior Frontal Gyrus		**↑**		DAN—FPN
R Uncus	L Inferior Frontal Gyrus		**↑**		LIM—FPN
R Lingual Gyrus	L Superior Temporal Gyrus			**↑**	VIS—DMN
R Posterior Cingulate	R Middle Occipital Gyrus		**↑**		Within VIS
L Middle Occipital Gyrus	R Cuneus		**↑**		Within VIS
R Precuneus	R Inferior Parietal Lobule			**↑**	VIS—VAN
R Middle Temporal Gyrus	R Superior Frontal Gyrus		**↑**		FPN—DMN
R Transverse Temporal Gyrus	R Angular Gyrus			**↑**	SMN—DAN
L Middle Frontal Gyrus	R Precentral Gyrus		**↑**		FPN—SMN

*DAN, dorsal attention network; DMN, default mode network; LIM, limbic network; FPN, frontoparietal network; hanblue, somatomotor network; VAN, ventral attention network, VIS, visual network. Coloring networks is based on Thomas Yeo et al. (2011) 7-network estimate. ↑ denotes increase, ↓ denotes decrease.

**Table 3:** Different connections found in alpha PLI networks across time (T0, T1, T2) and after pairwise comparisons (L denotes left hemisphere; R denotes right hemisphere).

**Different connections found in alpha PLI networks across time (T0, T1, T2)**					**RSNs**
L Fusiform Gyrus	L Inferior Frontal Gyrus				DMN—FPN
R Fusiform Gyrus	R Middle Occipital Gyrus				Within VIS
L Fusiform Gyrus	L Inferior Frontal Gyrus				DMN—FPN
L Middle Temporal Gyrus	L Inferior Frontal Gyrus				DMN—FPN
L Superior Temporal Gyrus	L Sub-Gyral (BA47)				DMN—FPN
L Fusiform Gyrus	L Middle Frontal Gyrus				DMN—FPN
L Middle Occipital Gyrus	R Cuneus				Within VIS
R Cuneus	L Cuneus				Within VIS
R Middle Temporal Gyrus	R Precentral Gyrus				DMN—SMN
R Middle Occipital Gyrus	L Cuneus				Within VIS
R Middle Occipital Gyrus	L Cuneus				Within VIS
R Anterior Cingulate	L Cuneus				LIM—VIS
L Parahippocampal Gyrus	L Angular Gyrus				VIS—DMN
L Superior Temporal Gyrus	L Angular Gyrus				Within DMN
R Superior Frontal Gyrus	L Supramarginal Gyrus				Within FPN
R Inferior Frontal Gyrus	R Precentral Gyrus				VAN—DAN
L Middle Temporal Gyrus	L Inferior Parietal Lobule				Within DMN
L Superior Temporal Gyrus	L Inferior Parietal Lobule				SMN—DMN
R Postcentral Gyrus	R Superior Parietal Lobule				SMN—FPN
L Middle Temporal Gyrus	L Inferior Parietal Lobule				DAN—FPN
L Middle Temporal Gyrus	L Inferior Parietal Lobule				DMN—FPN
L Superior Temporal Gyrus	L Inferior Parietal Lobule				DMN—FPN
L Superior Temporal Gyrus	L Inferior Parietal Lobule				SMN—FPN
L Superior Temporal Gyrus	L Inferior Parietal Lobule				SMN—FPN
L Middle Temporal Gyrus	L Inferior Parietal Lobule				DMN—FPN
L Inferior Frontal Gyrus	R Middle Frontal Gyrus				FPN—DMN
L Inferior Frontal Gyrus	R Middle Frontal Gyrus				Within DMN
R Anterior Cingulate	L Superior Parietal Lobule				LIM—FPN
L Middle Temporal Gyrus	L Superior Parietal Lobule				DMN—DAN
R Middle Temporal Gyrus	R Superior Parietal Lobule				DMN—FPN
L Middle Frontal Gyrus	L Inferior Parietal Lobule				DMN—DAN
L Sub-Gyral (BA47)	R Precentral Gyrus				FPN—SMN
L Middle Frontal Gyrus	R Precentral Gyrus				FPN—SMN
R Middle Temporal Gyrus	R Postcentral Gyrus				DMN—SMN
L Precentral Gyrus	R Middle Frontal Gyrus				VAN—FPN
L Inferior Frontal Gyrus	R Postcentral Gyrus				FPN—SMN
L Anterior Cingulate	R Postcentral Gyrus				DMN—SMN
L Sub-Gyral (BA47)	R Postcentral Gyrus				FPN—SMN
Pairwise comparisons: ↑ ↓					RSNs
		T0 vs. T1	T1 vs. T2	T0 vs. T2	
L Fusiform Gyrus	L Inferior Frontal Gyrus	↓			DMN—FPN
L Fusiform Gyrus	L Inferior Frontal Gyrus	↓			DMN—FPN
L Superior Temporal Gyrus	L Sub-Gyral (BA47)	↓			DMN—FPN
R Middle Temporal Gyrus	R Precentral Gyrus			↑	DMN—SMN
R Middle Occipital Gyrus	L Cuneus		↑		Within VIS
R Inferior Frontal Gyrus	R Precentral Gyrus			↑	VAN—DAN
L Superior Temporal Gyrus	L Inferior Parietal Lobule	↑			SMN—DMN
R Postcentral Gyrus	R Superior Parietal Lobule	↑			SMN—FPN
L Middle Temporal Gyrus	L Inferior Parietal Lobule	↑			DMN—FPN
L Superior Temporal Gyrus	L Inferior Parietal Lobule	↑			SMN—FPN
L Inferior Frontal Gyrus	R Middle Frontal Gyrus			↑	FPN—DMN
L Inferior Frontal Gyrus	R Middle Frontal Gyrus			↑	Within DMN
L Sub-Gyral (BA47)	R Precentral Gyrus		↑		FPN—SMN
L Middle Frontal Gyrus	R Precentral Gyrus		↑		FPN—SMN
L Inferior Frontal Gyrus	R Postcentral Gyrus		↑		FPN—SMN
L Sub-gyral (BA47)	R Postcentral Gyrus		↑		FPN—SMN

*DAN, dorsal attention network; DMN, default mode network; LIM, limbic network; FPN, frontoparietal network; SMN, somatomotor network; VAN, ventral attention network; VIS, visual network. Coloring networks is based on Thomas Yeo et al. (2011) 7-network estimate. ↑ denotes increase, ↓ denotes decrease.

**Table 4:** Different connections found in beta PLI networks across time (T0, T1, T2) and after pairwise comparisons (L denotes left hemisphere; R denotes right hemisphere).

**Different connections found in beta PLI networks across time (T0, T1, T2)**					**RSNs**
R Inferior Temporal Gyrus	R Anterior Cingulate				LIM—DMN
R Fusiform Gyrus	RAnterior Cingulate				LIM—DMN
L Inferior Occipital Gyrus	R Medial Frontal Gyrus				VIS—DMN
L Cingulate Gyrus	R Precentral Gyrus				DMN—SMN
R Superior Frontal Gyrus	L Superior Parietal Lobule				FPN—DAN
L Inferior Occipital Gyrus	L Sub-Gyral (BA47)				VIS—DAN
L Inferior Occipital Gyrus	L Sub-Gyral (BA47)				VIS—DAN
L Middle Occipital Gyrus	R Superior Frontal Gyrus				VIS—DMN
L Middle Temporal Gyrus	R Superior Frontal Gyrus				Within DMN
L Middle Occipital Gyrus	R Superior Frontal Gyrus				VIS—DMN
L Postcentral Gyrus	R Postcentral Gyrus				Within SMN
Pairwise comparisons: ↑ ↓					RSNs
		T0 vs. T1	T1 vs. T2	T0 vs. T2	
L Middle Occipital Gyrus	R Superior Frontal Gyrus	↑			VIS—DMN
L Middle Temporal Gyrus	R Superior Frontal Gyrus	↑			Within DMN
L Middle Occipital Gyrus	R Superior Frontal Gyrus	↑			VIS—DMN

*DAN, dorsal attention network; DMN, default mode network; LIM, limbic network; FPN, frontoparietal network; SMN, somatomotor network; VAN, ventral attention network; VIS, visual network. Coloring networks is based on Thomas Yeo et al. (2011) 7-network estimate. ↑ denotes increase, ↓ denotes decrease.

**Figure 8:**
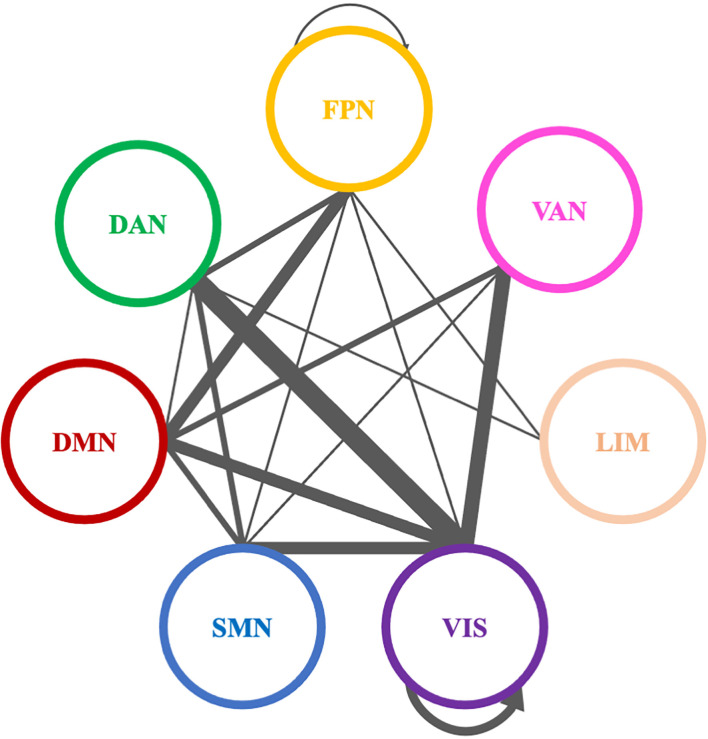
Significantly modified connections of theta PLI networks across time (T0, T1, T2), between or within the seven resting state networks (RSNs; Thomas Yeo et al., 2011). Line weight corresponds to number of significant connections. Most changes were observed in connections between visual network and other RSNs, between default mode network and frontoparietal network or within the visual network. DAN, dorsal attention network; DMN, default mode network; LIM, limbic network; FPN, frontoparietal network; SMN, somatomotor network; VAN, ventral attention network; VIS, visual network.

Comparing theta PLI networks across the evaluation phases 42 significant connections were revealed (Table 2). Most changes (27 out of 42) were observed in connections between VIS-DAN: seven (7), VIS-VAN: five (5), VIS-DMN: four (4), VIS-SMN: four (4), DMN-FPN: four (4), or within VIS: three (3) (Figure 8).

Pairwise comparisons revealed that six (6) out of 42 connections changed between T1 and T2, while three (3) out 42 connections were modified between T0 and T2 (Table 2); all those connections showed an increase in synchronization as measured by PLI. In that sense, seven (7) edges were observed to connect different RSNs (Table 2), with four (4) of them being between FPN and other RSNs, whereas two (2) edges were within the visual network (VIS).

**Figure 9:**
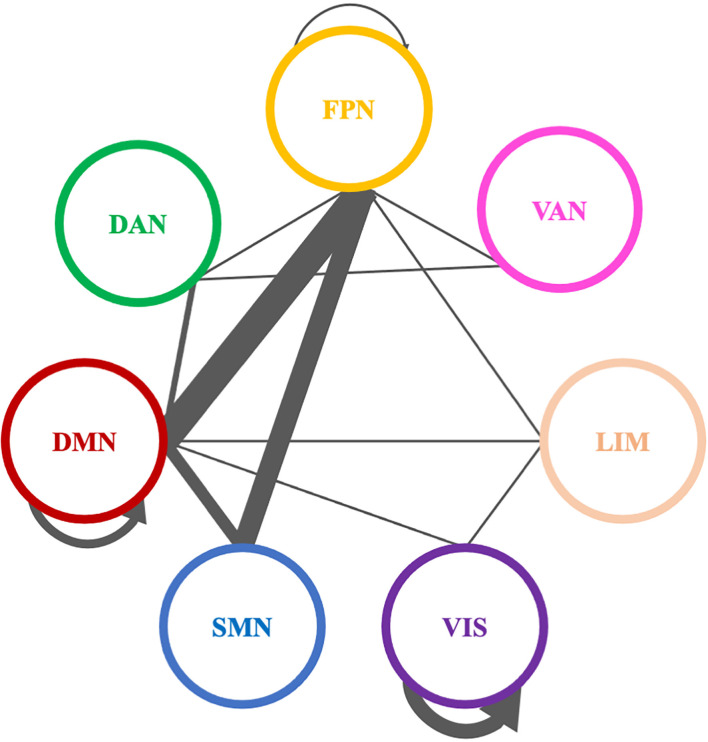
Significantly modified connections of alpha PLI networks across time (T0, T1, T2), between or within the seven resting state networks (RSNs; Thomas Yeo et al., 2011). Line weight corresponds to number of significant connections. Most changes were observed in connections between default mode network, frontoparietal network, and somatomotor network or within the default mode network and the visual network. DAN, dorsal attention network; DMN, default mode network; LIM, limbic network; FPN, frontoparietal network; SMN, somatomotor network; VAN, ventral attention network; VIS, visual network.

A total of 38 connections were significantly modified across time in alpha PLI networks (Table 3). Most changes (29 out of 38) were observed in connections between FPN-DMN: ten (10), FPN-SMN: seven (7), DMN-SMN: four (4), or within VIS: five (5) and DMN: three (3) (Figure 9).

Three (3) out of those 38 connections showed a decrease in PLI synchronization between T0 and T1 (all between DMN and FPN) and four (4) out of 38 connections showed an increase (see Table 3). Increase in synchronization was indicated in five (5) out of 38 connections between T1 and T2 whereas four (4) out of 38 connections showed an increase between T0 and T2. In summary, fourteen (14) connections emerging in pairwise comparisons were found between different RSNs involving mainly DMN, FPN, and SMN networks, while one (1) edge was observed within visual and one (1) within DMN networks respectively (Table 3).

**Figure 10:**
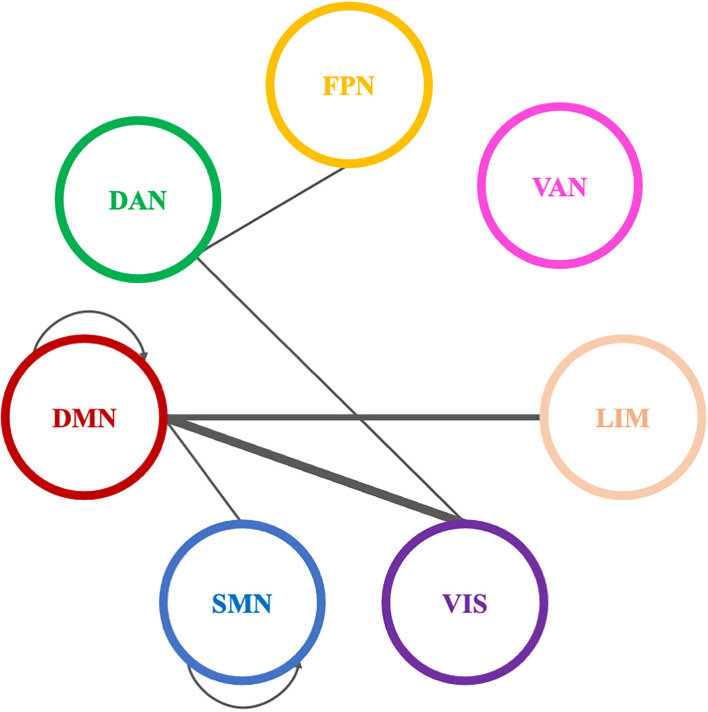
Significantly modified connections of beta PLI networks across time (T0, T1, T2), between or within the seven resting state networks (RSNs; Thomas Yeo et al., 2011). Line weight corresponds to number of significant connections. Most changes were observed in connections between default mode network and visual network or within the default mode network. DAN, dorsal attention network; DMN, default mode network; LIM, limbic network; FPN, frontoparietal network; SMN, somatomotor network; VAN, ventral attention network; VIS, visual network.

A total of eleven (11) connections were significantly modified in beta PLI networks across time (see Table 4; Figure 10). Three (3) of them were observed between VIS and DMN.

Pairwise comparisons revealed that three (3) out of 11 connections showed an increase between T0 and T1. Two (2) of them were between VIS and DMN and one (1) connection within DMN (Table 4).

##### 3.3.3.2 Graph properties showed a non-significant trend towards more efficiency and more distributed topology of theta networks

Possible alterations in different graph properties of PLI networks at theta, alpha, and beta band were explored. An increasing trend was observed in the clustering coefficient (CC) and global efficiency (EF) of theta PLI networks across time. A decreasing trend was shown in characteristic path length (CPL) of theta PLI networks across time. Alterations in alpha PLI network properties across time were not revealed even though a decreasing trend was shown in CPL. Furthermore, we did not observe any modifications in the graph properties of beta PLI networks across time.

## 4 Discussion

### 4.1 Combined BF/NF training improved smoking status and played a protective role with regards to smoking-induced oxidative load

Exhaled CO levels constitute a non-invasive, inexpensive and direct biochemical method to evaluate smoking status (Deveci et al., 2004; Javors et al., 2005; European Network for Smoking Prevention, 2018; Benowitz et al., 2020). The updated report on biochemical verification of cigarette smoking (Benowitz et al., 2020) proposed the use of a CO cutoff at 6 ppm (Emery and Levine, 2016) when evaluating the effectiveness of smoking cessation interventions. Multiple factors may affect exhaled CO levels such as air pollution levels (Gregorczyk-Maga et al., 2019), exercise, minute ventilation, and type of CO monitor used. Participants in our study showed decreased CO levels across time. Similar findings were observed in low-dependent/moderate smokers. CO levels lower than 6 ppm (successful quitters) were not measured at T0, while they were found in 2 out 17 (11.76%) at T1 and in 4 out 17 smokers (23.53%) at T2. All successful quitters at the end of the intervention, initially were low-nicotine dependent smokers, while their proportion is comparable to other smoking cessation strategies (Woolacott et al., 2002). Moreover, a decrease in TOS levels was found among the participants. Smoking is characterized by increased oxidative damage and declined antioxidant defense (Kamceva et al., 2016; Karademirci et al., 2018) leading to free radicals generation and inflammatory response (Csordas and Bernhard, 2013; Messner and Bernhard, 2014) that affect tissue, lipids, and genome integrity (Halliwell, 1987; Benowitz and Gourlay, 1997; Karademirci et al., 2018), while a correlation exists between oxidative stress with the number of cigarettes smoked (Kamceva et al., 2016). A protective role of BF/NF training against smoking-induced oxidative load could be argued, as in our study severe smokers/high-nicotine dependent individuals (who did not quit smoking) also achieved a significant TOS reduction across time. This was also supported through reducing nicotine dependence in severe smokers, as measured by Fagerström scores across time, which is one of the most widely recognized and used tools for measuring nicotine dependence (Haighton et al., 2013; Engelmann et al., 2020).

Furthermore, smoking “attacks” the immune system by depleting vitamin E and other antioxidants (Eiserich et al., 1995; Handelman et al., 1996; Leonard et al., 2003; Bruno and Traber, 2006). However, some studies supported that vitamin E plasma status is preserved in smokers (Lykkesfeldt et al., 2000; Dietrich et al., 2003), proposing the presence of regulatory mechanisms [currently largely unknown (Bruno and Traber, 2006)] that lead to compensation of plasma vitamin E concentration despite its increased metabolism (Traber et al., 2001; Bruno and Traber, 2006). Moreover, ascorbic acid (vitamin C) is negatively correlated with the rate of vitamin E clearance and seems to preserve its antioxidant function (Bruno et al., 2005). In our study, Vitamin E levels significantly increased across time (T0, T1, T2) in males, while between T0 and T2 in females. This gender-related pattern could be attributed to differences in biochemical and hematological indices or food choices leading to variation in nutrients intake (Bates et al., 1999). Additionally, elevated levels were shown across time and between T0 and T2 in low-dependent/moderate smokers, across time in high-dependent individuals and in the whole group of participants between T0 and T2. Vitamin E is also known for its neuroprotective properties (Khanna et al., 2005; González-Fuentes et al., 2018) and the increase that we observed may as well partly explain or complement the neuroprotective role of BF/NF training, that we discussed in our previous work (Pandria et al., 2018), as well as the very significant gains in behavioral profiles of the participants that we discuss below.

### 4.2 Combined BF/NF training faded out anxiety, enhanced self-esteem, and improved aspects of cognitive performance

The inter-relationship between smoking and affective state was early described as “Nesbitt’s paradox” (Nesbitt, 1973) characterizing smoking as both stimulant and sedative. What many researchers accepted as a paradox (Dunn, 1978; Gilbert, 1979; Pomerleau and Pomerleau, 1991, 2007), Parrott (1998) described as the deprivation reversal model, in which smoking is interpreted as a normalizing factor. As such smoking promotes positive alterations (relaxation, alertness) through nicotine replenishment which reverse the negative effects of abstinence (irritability, anger, nervousness, anxiety, increased appetite, poor task performance and concentration, depression, insomnia; Manos, 1997; American Psychiatric Association, 2013). Moreover, smoking causes dysregulation of common hormonal stress responses and prolonged discomfort state (Childs and de Wit, 2009) supporting the establishment of abnormal anxiety and arousal thresholds. Mood deviation in long and short abstinence periods (e.g., between two cigarettes) leads to conditioned learning (Kalantzi-Azizi and Degleris, 1992; Parrott, 1998). In that sense, abstinence and other negative affective states could be interpreted as stressors leading to smoking, precluding abstinence and relieving the ongoing negative affective state (Parrott, 1998). A correlation between relapse and stress is well established as many smokers (ranging from 35% to 100%) lapses while experiencing negative affective states or stressful conditions (Shiffman, 1982; Cummings et al., 1985; Borland, 1990; Brandon et al., 1990). Therefore, stress constitutes a core element implicated in all stages of nicotine addiction as described in the modified version of Flay’s stage model (Pandria et al., 2018).

Considering that stress could be a mediator factor of maintaining the vicious cycle of nicotine abstinence, we designed a multi-visit BF/NF investigation that both implemented tools of stress alleviation and studied related behavioral outcomes. In our study, positive modifications to stress were observed as State-Trait Anxiety Inventory scores decreased over time in the pool of participants. Greater improvement was observed in prolonged anxiety symptoms (Trait Anxiety scores) between T0 and T2. Similar outcomes were especially revealed in low-dependent/moderate smokers. The smoking cessation process is characterized by reduced anxiety, stress, and depression as well as improvements in mood and quality of life in relation to non-quitters (Taylor et al., 2014) and the observed improvements in anxiety and other behavioral aspects in our participants indicate this process.

This suggestion may be further supported by improved self-esteem (Rosenberg, 1965; Baumeister et al., 2003) that was observed in the pool of participants and particularly in females and high-dependent individuals. Self-esteem is considered a motivational factor (West and Brown, 2013) for adopting behaviors that improve and protect wellbeing and health status (Wellman et al., 2016; Du et al., 2017). Although the negative correlation between self-esteem and smoking is under investigation (Szinay et al., 2019), high self-esteem leads to a greater possibility to quit smoking (Freijy and Kothe, 2013). To that sense, participants in the BF/NF training reported higher self-esteem that could lead to a greater chance of quitting smoking. Considering that females are characterized by higher perceived stress, decreased self-esteem and lower body image satisfaction (Croghan et al., 2006), marginally significant gains in self-esteem observed in females but not in males strengthens the role of BF/NF training. Similarly, moderate smokers or high-nicotine dependent individuals have been characterized by low self-esteem (Guillon et al., 2007). Combined training seems to increase the perceptions and beliefs about self-worth in high-dependent smokers, especially between T0 and T2, but this did not translate to a successful smoking cessation outcome for this group.

The deprivation reversal model (Parrott, 1998), or negative reinforcement (Valentine and Sofuoglu, 2018), seems also applicable in cognitive performance. Nicotine may promote cognitive enhancement by stimulating cognitive-related brain areas such as prefrontal cortex and hippocampus (Wallace and Bertrand, 2013; Kutlu and Gould, 2015) and by facilitating synaptic plasticity in significant neural circuits (Couey et al., 2007) through specific subunits of nicotine acetylcholine receptors (nAChRs; Kenney and Gould, 2008). However, there is no clear evidence that nicotine is genuinely cognitive-enhancing as cognitive empowerment could be a manifestation of withdrawal relief (Heishma et al., 1994; Heishman, 1998). Moreover, smoking abstinence is characterized by impaired attention, working memory and concentration (Parrott et al., 1996; Xu et al., 2005; McClernon et al., 2008; Harrison et al., 2009). Cognitive manifestations of abstinence seem to be reversed either by re-exposing deprived smokers to nicotine or by providing smoking cessation treatment (Davis et al., 2005; Portugal and Gould, 2007; Patterson et al., 2009).

In the combined BF/NF intervention, participants showed improved inhibition control (as assessed by total Stroop scores) across time, with cognitive gains mainly found between T0 and T2. Similar findings were observed in females and low-dependent/moderate smokers. High-dependent smokers showed higher total Stroop scores across all evaluation stages (T0, T1, T2). Moreover, severe smokers/high-dependent individuals showed improved visual attention and task-switching (as assessed by Trail A test) and better working memory (as evaluated by Digit Span total scores) across time. As cognitive deficits have also been associated with relapse after smoking cessation (Ashare et al., 2014), the cognitive enhancement that we observed after BF/NF training could provide cues for a preventive role against relapse, something which should be evaluated across long time periods.

### 4.3 Learning effects were validated in longitudinal electrophysiological data

#### 4.3.1 Feedback learning was achieved within sessions in BF training as average temperature significantly increased from baseline

Grimsley (Grimsley, 1990) first introduced skin temperature training on smokers taking into account smoking behavior (abstinence or cigarette consumption) prior to BF training. In his study, deprived smokers showed higher baseline temperature compared to never smokers and non-deprived smokers but they did not manage to increase their skin temperature after training (Grimsley, 1990). Greater enhancement in skin temperature was achieved by never smokers, followed by smokers who abstained for at least 1 h. Physiological responses to smoking and nicotine-induced vasoconstriction plays a role in this pattern (Benowitz and Burbank, 2016). Participants in our study successfully increased their temperature in all sessions (apart from the first session) compared to the corresponding baseline. The failure to increase skin temperature during the first session could be attributed to familiarization process with the methods of BF training (Gruzelier et al., 2010). The findings were driven by low-dependent/moderate smokers, as high-dependent/severe smokers showed significant positive temperature alterations only in one session (3th). This better performance by low dependent/moderate smokers can be attributed to lower nicotine intake as nicotine negatively affects training (Griffith and Crossman, 1983; Szalai et al., 1986; Grimsley, 1990).

Gender differences in skin temperature are well established as females are characterized by lower skin temperature of the trunk, the upper and lower extremities compared to males (Neves et al., 2017). Subcutaneous fat (Neves et al., 2017) and menstrual cycle are among the parameters involved that affect temperature (Nagashima, 2015). Nonetheless, differences based on gender grouping were not observed in this aspect of our study, as female smokers increased their temperature in two out of five sessions (4th and 5th) while males in three sessions (2nd, 3rd, and 5th).

Carry-over learning across pre-training baseline was not observed for any grouping. A possible explanation could be the continuation of participants’ smoking habits during the first stage (BF) of intervention, that we also reported in our previous work (Pandria et al., 2018). Regarding the interpretation of the above-mentioned temperature patterns in terms of feedback learning, guidelines for reporting BF learning do not exist, to the best of our knowledge. Thus, BF learning was approached based on forms of learning adopted by NF research (Gruzelier, 2014c).

#### 4.3.2 Feedback learning was observed in theta/alpha ratio in NF training within sessions, while carry-over learning was observed across session baselines

Peniston and Kulkosky (1989) first introduced the alpha-theta protocol for war veterans suffering from PTSD and alcohol abuse (Peniston and Kulkosky, 1991, 1993). Their work focused on theta over alpha enhancement at occipital brain regions after applying skin temperature training and breathing exercises. They observed positive outcomes mainly in personality variables, dosage of medication, prolongation of abstinence, and prevention of relapses (Peniston and Kulkosky, 1989, 1991, 1993). Although the precise mechanisms of alpha-theta training remain elusive (Gruzelier and Egner, 2005), two possible explanations were formulated: (1) training leads to a cross-over state (theta exceeds over alpha rhythm) which contributes to psychological integration (Peniston and Kulkosky, 1993) and (2) counteracting the increase in beta-endorphin levels induced by stress of abstinence (Peniston and Kulkosky, 1989). A condition that was termed “Peniston flu” was described, implying that a considerable portion of addicted individuals experienced flu-like symptoms against the substance of their abuse (Demos, 2005). Due to its high success and acceptance, alpha-theta protocol was applied to other substance abuse disorders (Sokhadze et al., 2008) or was modified to be used in polysubstance abusers (Scott and Kaiser, 1998; Scott et al., 2005).

To the best of our knowledge, this is the first study which applies a modified multi-modal/multi-visit version of alpha-theta protocol on nicotine addiction. Exploring NF learning of the trained index (theta/alpha ratio), we observed that participants achieved increase of baseline ratio across sessions (carry-over learning; Gruzelier, 2014c). Moreover, theta/alpha ratio seems to be enhanced across baseline in low-dependent/moderate smokers but not in high-dependent/severe smokers. A possible explanation could be found in the suggestion of Canterberry et al. (2013) that low-nicotine dependent individuals may more effectively modify craving-related brain activity compared to high-dependent smokers. This suggestion is further supported by the greater vulnerability to withdrawal that characterizes highly-dependent smokers. Thus, they have difficulty in abstaining from smoking for more than a few hours regardless of the motive for smoking cessation (Shiffman et al., 2006).

Moreover, a significant increase in theta/alpha ratio was found within sessions both in comparisons of baseline to session average and in comparisons of baseline to after training measurements (successful within session learning). As we previously stated, carry-over learning of baseline ratio was observed, however, carry-over learning of session average ratio was not observed. This finding is quite expected as in low arousal states (i.e., in alpha-theta training) it is more possible to reveal feedback learning within sessions than across sessions (Gruzelier, 2014c). A probable interpretation could be that the characteristics of theta/alpha modulation (such as magnitude and depth) tend to fluctuate depending on the state of the subject during training (Gruzelier, 2014b, c). Similarly, significant alterations in theta and alpha amplitude respectively were shown only within sessions. Greater gains in alpha-theta learning seem to be in favor of low-dependent/moderate smokers and females considering within session comparisons too. Although the possible role of nicotine dependence has already been discussed, gender differences in feedback learning have not yet been explored, nor the impact of menstrual cycle (Muravleva et al., 2012; Gruzelier, 2014c).

### 4.4 Organization of resting state PLI networks was positively modified across time

#### 4.4.1 VIS, DMN, and FPN, that are highly involved in nicotine addiction, demonstrated positive functional reorganization across time

We provide evidence that our group of smokers presented capacity for neuroplasticity. Combined BF and NF training altered the communication within and between nodes of the core RSNs (Thomas Yeo et al., 2011). Specifically, the altered connectivity across time (T0, T1, T2) within and between the core RSNs (Thomas Yeo et al., 2011), underscores the post-intervention functional reorganization of the brain of the study’s subjects. While only a limited number of studies have investigated RSNs with a specific focus on tobacco chronic use (Weiland et al., 2015), the current study investigates their reorganization following an alternative (non-pharmacological) smoking cessation approach. Given the theoretical proposal that nicotine addiction manifests as a dysfunction in the dynamic connections between large-scale brain networks (Sutherland et al., 2012), the group of smokers who completed our intervention exhibit increased synchronization mainly between the networks that support cognitive control (FPN), goal-directed behavior (DMN), and visual processing (VIS), as well as within the VIS and the DMN. These networks have previously been implicated in substance abuse (Sutherland et al., 2012) and neurocognitive and rewarding responses to nicotine, while failure to control inhibition may contribute to the addiction cycle (Goldstein and Volkow, 2002).

Nicotine binds to nicotinic acetylcholine receptors, found in many of the regions included in the VIS, FPN, and DMN (Mamede et al., 2004; Naser et al., 2012). Chronic nicotine use impacts the connectivity of these networks negatively, which in turn plays a role in the difficulty smokers experience in quitting (Weiland et al., 2015). Vergara et al. (2017) distinguished visual and sensorimotor regions as areas whose resting state connectivity is decreased by chronic nicotine use. Tanabe et al. (2011) observed that chronic nicotine use suppresses the connectivity within DMN in the absence of an external demanding task. Moreover, Musso et al. (2007) observed that prefrontal networks involved in attention show diminished activity correlating with smoking duration. On the contrary, it has been found that increased activity within the DMN is related to abstinence from smoking and it could be interpreted as a compensatory mechanism in order to maintain homeostasis during the abstinence phase (Sutherland et al., 2012). We should point here that, in our investigation, three connections between DMN and FPN showed a decrease in alpha rhythm synchronization after the BF sessions, while all other significant pairwise changes, including those within DMN and within VIS corresponded to increase in synchronization, showing an overall beneficial effect on these networks’ communication. Furthermore, increased synchronization between VIS and other RSNs [including VAN as a carrier of salience (Thomas Yeo et al., 2011)], a prominent finding in our investigation, is considered an indication of better sensory information integration (Dobrushina et al., 2020). Finally, Modi et al. (2015) found that Trait anxiety is associated with reduced connectivity in DMN and VIS, among others. In our study we found reduction in Trait anxiety between T0 and T2, that could reflect to our observed increase within VIS and DMN. We theorize that the altered connectivity across time evidenced here is related to the improvement in the smoking status and behavioral profile of our group, which is supported by the general harm reduction achieved, through significant decrease of exhaled CO, TOS, and Fagerström score, as well as through an increase of Vit E levels.

#### 4.4.2 Theta and alpha PLI networks synchronization was increased after NF training while beta band resting state networks mostly changed significantly after BF

In support of our theoretical proposal is that alpha-band RSNs exhibit mainly increased between connectivity pairwise between all three time points (T0 vs. T1, T1 vs. T2, and T0 vs. T2), theta band RSNs show increased between connectivity in both T1 vs. T2, and T0 vs. T2, while beta band RSNs change significantly only in T0 vs. T1. Changes in body temperature and arousal levels (BF training T0 vs. T1) can cause changes in alpha and beta bands by hypothalamic modulation (Nybo and Nielsen, 2001). Considering the alpha-theta NF training our subjects received between T1 and T2 and its implication in addictions other than smoking (Peniston and Kulkosky, 1989; Masterpasqua and Healey, 2003; Scott et al., 2005; Sokhadze et al., 2008), post-traumatic stress disorder (Peniston and Kulkosky, 1991) and mood improvement (Raymond et al., 2005a), it is plausible that extended reorganization of alpha and theta PLI networks (i.e., increase in alpha and theta synchronization) hints the efficacy of this alternative training for smoking cessation. We also provided indications, through increased synchronization within DMN and between DMN and other RSNs that our group of participants engaged in abstinence from smoking. Future studies should further assess our suggestion to determine how our results fall into place with previous studies reporting that nicotine abstinence causes increases in EEG power in low-frequency bands (delta and theta) and leads to reductions in high-frequency bands (alpha and beta) during a resting state (Teneggi et al., 2004).

#### 4.4.3 Stability of whole brain resting state topology is indicative of countering expected negative effects of smoking in brain network properties

Possible alterations in whole brain RSN graph properties of theta, alpha, and beta band were explored but did not produce significant results, showing a stability of key organization properties of brain topology (Rzucidlo et al., 2013). We also described this finding in our previous work (Pandria et al., 2018) where we did not find changes between T0 and T1. FCN topology has been demonstrated to be affected in smoking, associated with severity and duration, to the direction of lower global efficiency and greater path lengths (Lin et al., 2015). Smoking-induced chronic stress may play a role and stress-related disorders presenting similar alterations in RSN topology (Akiki et al., 2018). Nonetheless, in our work, we describe a non-significant trend towards more efficiency (increased global efficiency and decreased characteristic path length) and more segregated or distributed topology (increased clustering coefficient) of whole brain theta RSN. A non-significant trend towards more efficiency (decreased characteristic path length) was also observed in the alpha rhythm networks, while no trends were observed in beta rhythm networks. These trends were not observed in our previous analyses either (Pandria et al., 2018). These results fall into place within our theoretical proposal, as they indicate that the trained NF index (theta/alpha ratio) may positively affect RSN topology organization countering expected negative effects of stress and smoking habits to global brain resting state topological features (Fedota and Stein, 2015). The combined intervention, as such, is maybe shown to play an enhanced neuroprotective role, complementary to the physical manifestations of reduced oxidative load and increased Vitamin E that we previously described.

## 5 Importance, limitations and future perspectives

Tobacco epidemic causes premature death and multi-level morbidity worldwide (World Health Organization, 2019) highlighting the necessity of tailored actions for smoking cessation. Although the World Health Organization (WHO) encouraged countries to adopt the WHO Framework Convention on Tobacco Control (World Health Organization, 2021), smoking cessation is still challenging. Education, behavioral interventions, and pharmacotherapy are considered to be the ingredients of a successful cessation attempt (Fiore et al., 2008; European Network for Smoking Prevention, 2018). Moreover, non-pharmacological regimes have been developed to address the needs of specific populations (i.e., pregnant smokers, patients affected by acute coronary syndrome, severe mental illness or seizures) for whom pharmacotherapy could not be the treatment of choice (Rigotti, 2015; Siu and Preventive Services Task Force, 2015; ACog Committee Opinion, 2017). For all smokers, an important aspect of smoking cessation interventions is harm reduction, namely the reduction of adverse physical and mental health effects caused by tobacco use in smokers (McNeill, 2004; Taylor et al., 2014; West et al., 2015), through gradual reduction of smoking habits (McNeill, 2004; Lindson et al., 2019). In our study, we demonstrated clear benefits in reduction of nicotine use (through CO levels), in clinical status (through TOS and Vitamin E) in behavioral status, and in aspects of cognitive performance. Therefore, while the intervention has an acceptable smoking cessation ratio to other strategies (Woolacott et al., 2002), it also clearly demonstrates its importance towards harm reduction. Moreover, the intervention spanned 15 weeks, allowing time for gradual adjustment to the attempt (Kim et al., 2019; Lindson et al., 2019) and for coping with stress induced by failure to quit smoking abruptly or completely (Kim et al., 2019), as well as for longitudinal measurements of clinical, behavioral, and neurophysiological parameters.

Among classical BF/NF studies, Peniston protocol was applied in drug abuse, PTSD, substance abuse disorders (Sokhadze et al., 2008), and polysubstance abuse (Scott et al., 2005; Shiffman et al., 2006) with positive outcomes in prolongation of abstinence and prevention of relapses, among others. To the best of our knowledge, this is the first study which applies a modified multi-modal/multi-visit version of alpha-theta protocol on nicotine addiction. Regarding the implementation of the protocol, it should be also noted that individualized approaches to standardized NF protocols have been suggested, and tested, in order to improve training and clinical outcomes in conditions like Attention Deficit Hyperactivity Disorder (ADHD; Escolano et al., 2014; Bazanova et al., 2018), functional pain (Bazanova and Aftanas, 2010), and cognitive decline in elderly (Angelakis et al., 2007). Individual variability of brainwaves, starting with determination of individual alpha frequency (IAF), were taken into account with regards to peak alpha performance and alpha training (Angelakis et al., 2007; Bazanova and Aftanas, 2010; Escolano et al., 2014) and also theta/beta ratio (Bazanova et al., 2018). To the authors’ best knowledge, no study has applied individualized alpha-theta protocol yet for any condition. Nonetheless, it has been shown that individual differences in theta and alpha dynamics correlate with functional hemodynamic brain responses during working memory (Meltzer et al., 2007) and reflect performance in cognitive and memory tasks (Klimesch, 1999).

Most recent NF studies that investigated changes in brain plasticity (Canterberry et al., 2013; Hanlon et al., 2013; Li et al., 2013; Kim et al., 2015; Hartwell et al., 2016; Bu et al., 2019) induced by training, were focused on response modulation to craving. Although investigation in brain modulation following NF training seems plausible, chronic exposure to nicotine affects almost all body organs (World Health Organization, 2019). Therefore, BF/NF protocols that aim to stand as an alternative or complementary aid to smoking cessation should be evaluated considering multiple clinical, biochemical, and behavioral measures in the course of time. In our work, training-induced feedback learning was approached based on forms of learning adopted by NF research Gruzelier (2014c) even though reporting guidelines are at an early stage. Training outcomes were interpreted in the light of modulation of RSNs networks. This is the first experimental study where the BF/NF protocol was evaluated using a multi-parametric approach.

Although we extensively presented the promising outcomes of a multi-modal approach in nicotine addiction, we should take into consideration several limitations of our study. The high-dropout rate was a common issue among SmokeFreeBrain project partners. Reported reasons for drop-out from our study included personal reasons (e.g., moving abroad), medical conditions (e.g., manifestation of cancer), and negative experiences during EEG recordings (e.g., claustrophobia, discomfort). A number of obstacles that were considered also included poor experimental protocol adherence, complains of time-consuming procedures (physical presence required), and delayed training benefits. The consequence of the high dropout rate was a limited sample size of 17 smokers that completed the multi-visit intervention and all three evaluation phases, out of an initial cohort of 49 recruited participants. However, it should be underlined that every subject completed three evaluation stages (three EEG recordings among others) along with a 25-session training (30–40 min/each session) intervention. As such, we gathered data from a total of 425 feedback sessions and a total 476 electrophysiology sessions across 15 weeks. The aforementioned issue is discussed by Gruzelier (Gruzelier, 2014a) when referring to the limited number of subjects that are typically recruited in NF studies, suggesting that large datasets cope with this limitation. It should note that many BF and NF investigations on smoking are underpowered, with *N* < 10 (Griffith and Crossman, 1983; Canterberry et al., 2013; Li et al., 2013; Hanlon et al., 2013).

Another limitation of our study can be identified in the lack of a control group (Ros et al., 2020); however, we adopted a prospective longitudinal design tracking the progress of our participants over time using repeated measures of the same variables. Moreover, the lack of a comparator group is common among published BF and NF investigations on nicotine addiction (Griffith and Crossman, 1983; Canterberry et al., 2013; Hanlon et al., 2013; Li et al., 2013; Pandria et al., 2018). We may also identify relative gender imbalance in our sample, as 64.7% of participants were female, that constricted between-group analysis of gender effects. Nonetheless, the limited literature on BF and NF intervention for smoking (Pandria et al., 2020) shows that these studies chronically suffer from gender imbalance, much more commonly in the direction of including more male subjects (Griffith and Crossman, 1983; Kim et al., 2015; Bu et al., 2019). As female smokers are underrepresented in these studies, while in some countries they are comparable portions of smoker populations to male smokers (World Health Organization, 2020), it is important to study and validate smoking cessation interventions considering gender-related patterns.

In our future work, we aim to investigate the characteristics of participants who dropped out of the study against those who completed the multi-visit intervention. Segmentation of continuous EEG measurements during NF sessions will be analyzed as intra-session deviations are highly dependent on the task or the mental strategy used during separate periods of training (Dempster and Vernon, 2009; Gruzelier, 2014c). Longitudinal changes in clinical, biochemical, and behavioral indices will be compared between the BF/NF multi-visit study and a pharmacological study implemented in the context of SmokeFreeBrain project. Future follow-up interventions will be designed also taking into account IAF variability.

## 6 Conclusions

Combined BF/NF training positively affected the clinical and behavioral status of smokers and displayed clear benefits towards harm reduction. The intervention improved smoking status and played a protective role with regards to smoking-induced oxidative load. Moreover, the training protocol resulted in positive modifications in anxiety, enhanced self-esteem, and improved aspects of cognitive performance. Low-nicotine dependent and moderate smokers especially showed more benefits compared to high-nicotine dependent and severe smokers, while no clear outcome was observed with regards to gender grouping. Combined BF/NF training lead to neuroplastic learning effects, that were validated in longitudinal electrophysiological data, and leads to reorganization of RSNs across time. Feedback learning was achieved within sessions in BF training as average temperature significantly increased from baseline measurements. Moreover, feedback learning was observed in theta/alpha ratio in NF training within sessions, while carry-over learning was observed across session baselines. We provided evidence that our group of smokers presented capacity for neuroplasticity. PLI network connections were significantly modified across time mainly in theta and alpha rhythms, while beta band RSNs mostly changed significantly after BF. Significantly altered connections were mapped either uniquely within a RSN or between different RSNs according to the 7-netwok estimate. Limitations of our study include high-drop rate and lack of comparator group, common among BF/NF studies, that hamper generalization of our results.

## Data availability statement

The datasets presented in this study can be found in online repositories. The names of the repository/repositories and accession number(s) can be found in the article/Supplementary material. Data from the SmokeFreeBrain project (http://smokefreebrain.eu/) are accessible in the following link: http://ckan.smokefreebrain.eu/search/type/dataset under an Attribution NonCommercial NoDerivatives 4.0 International license. Data of the analysis presented in the current manuscript and the related code can be made available via a request of the authors following a Memorandum of Understanding (MoU) in the context of Open Research Initiative.

## Ethics statement

The studies involving human participants were reviewed and approved by Ethics and Bioethics Committee of the School of Medicine at the Aristotle University of Thessaloniki. The patients/participants provided their written informed consent to participate in this study (meeting no. 4/2-6-2016 with protocol number: 316/4-7-2016).

## Author contributions

PB conceptualized the initial idea that was shaped as an intervention into the SmokeFreeBrain project. NP organized the creation of this work. NP, EP, and PB wrote the study protocol. NP, AA, CS, EP, and MK conducted the experiments. NP conducted all bio/neurofeedback sessions. MK and EP conducted neuropsychological assessments. AP and CK-P conducted clinical assessments. NP and NT conducted electrophysiological and statistical analyses. KMa, SI, and EL conducted biochemical analyses. NP and AA wrote the introduction, methods, and results. NP, AA, and CS interpreted the results, drafted, and proof-read the manuscript. KMi contributed to tables and figures and Supplementary material. PB contributed to shaping the content of the article, drafting, and proofreading the final submitted version of the manuscript and supervised the complete writing process. All authors contributed to the article and approved the submitted version.

## Footnotes

^1^ https://clinicaltrials.gov/ct2/show/NCT02991781
