# Supplementary material for: Does combined training of biofeedback and neurofeedback affect smoking status, behavior, and longitudinal brain plasticity?
Source: Front Behav Neurosci. 2023 Jan 27;17:1096122. doi: 10.3389/fnbeh.2023.1096122 (PMC9911884; doi:10.3389/fnbeh.2023.1096122)
Supplement: Supplementary file 1 [file Data_Sheet_1.docx]

Supplementary Material

# Statistical analysis results in detail

In this document, submitted as supplementary material [S1] to the manuscript titled “**Does combined training of biofeedback and neurofeedback affect smoking status, behavior and brain plasticity?**”, we included the detailed results from the statistical analysis of the collected data. The inclusion of these results in the main manuscript was deemed disrupting for its readability so we decided to provide them separately.

[1 Statistical analysis results in detail 1](#_Toc1629611081)

[1.1 Clinical Data 2](#_Toc1951474743)

[1.1.1 Exhaled Carbon Monoxide (CO) 2](#_Toc1053817167)

[1.1.2 Spirometric parameters (FEV1%, FVC%, FEF25-75%) 2](#_Toc583100094)

[1.1.3 TOS and vitamin E 2](#_Toc2077953952)

[1.2 Behavioral Data 3](#_Toc1349248670)

[1.2.1 Self-administered questionnaires 3](#_Toc1019382788)

[1.2.2 Neuropsychological assessment 6](#_Toc2134791845)

[1.3 Electrophysiological Data 7](#_Toc841416788)

[1.3.1 Biofeedback 7](#_Toc2086501997)

[1.3.1.1 Across baseline 8](#_Toc357734471)

[1.3.1.2 Baseline vs. Corresponding session 8](#_Toc556758890)

[1.3.1.3 Across sessions 8](#_Toc590256679)

[1.3.2 Neurofeedback 8](#_Toc1421493121)

[1.3.2.1 Across baseline 9](#_Toc1514134178)

[1.3.2.2 Baseline vs. Corresponding session 9](#_Toc439510395)

[1.3.2.3 Across sessions 17](#_Toc430289135)

[1.3.2.4 Pre-post sessions 18](#_Toc2067643234)

[1.3.3 EEG 24](#_Toc2059380336)

[1.3.3.1 Graph properties 24](#_Toc319559146)

[2 Acknowledgement 24](#_Toc555625057)

## Clinical Data

### Exhaled Carbon Monoxide (CO)

Exhaled CO measurements and estimated levels of carboxyhemoglobin (COHb) were considerably reduced across time conditions (T0, T1, T2) (CO - T0: 15.00, [8.50, 20.00]; T1: 13.00, [7.50, 23.00]; T2: 10.00, [5.50, 16.50]; χ^2^(2)=8.222; p=0.016; COHb - T0: 3.03, [1.99, 3.67]); T1: 2.71, [1.84, 4.31]; T2: 2.55, [1.51, 3.32]; χ^2^(2)=7.875; p=0.019). Although exhaled CO and COHb levels were found to be decreasing across T0 and T2 (CO: W=-2.039; p=0.041; p-corrected=0.123; COHb: W=-1.991; p=0.046; p=0.138), said changes were not retained, as a result of Bonferroni correction. Both female and male participants did not exhibit modified exhaled CO and COHb levels over time (CO - Female: χ^2^(2) =4.439; p=0.109; Male: χ^2^(2) =4.455; p=0.108; COHb – Female: χ^2^(2) =4.439; p=0.109; Male: χ^2^(2) =3.739; p=0.154).

A significant decrease in CO and COHb levels was observed in moderate smokers (CO – T0: 13.00, [8.00, 15.00]; T1: 11.00, [6.00, 18.00]; 8.00, [3.00, 13.00]; χ^2^(2) =7.951; p=0.019; COHb – T0:2.71, [1.91, 3.03]; T1:2.39, [1.59, 3.51]; T2: 1.91, [1.20, 2.71]; χ^2^(2) =7.429; p=0.024), but not for severe smokers (CO/COHb: χ^2^(2) =1.091; p=0.580). Similar outcomes were documented when grouping participants based on pack-years. Low-dependent smokers significantly reduced their CO and COHb levels across time (CO – T0: 11.50, [8.00, 15.75]; T1: 9.50, [5.75, 14.25]; 7.50, [3.00, 12.25]; χ^2^(2) =9.389; p=0.009; COHb – T0:2.47, [1.91, 3.15]; T1:2.16, [1.55, 2.91]; T2: 1.83, [1.18, 2.59]; χ^2^(2) =8.486; p=0.014). Changes in CO/COHb levels across time were not observed in high-dependent smokers (CO/COHb: χ^2^(2) =1.852; p=0.396).

### Spirometric parameters (FEV1%, FVC%, FEF25-75%)

After investigating changes in spirometric measures across time conditions (T0, T1, T2) we did not observe any alteration in all predicted measures (FEV1%: F(2,32) =2.077; p=0.142; FVC%: F(2,32) =2.333; p=0.133; FEF25-75%: F(2,32) =0.949; p=0.398). Both female and male participants did not show any change in spirometric measures across time conditions (T0, T1, T2). We observed, however, a marginally significant decrease in FVC% across T1 and T2 (T1:88.182±15.80; T2: 77.364±17.16; t(10) =-2.853; p-corrected=0.051). Splitting data based on their Fageström score, we did not find any considerable alteration in all the predicted measures for both groups (moderate, severe smokers) (all p-values>0.05). Nonetheless, we observed a significant decline in FVC% across time conditions (T0, T1, T2) in high-dependent participants (F(2,12) =7.690; p=0.007) but not in low-dependent subjects after grouping data with respect to pack-years. Pairwise comparisons revealed that FVC% was decreased across T1 and T2 (T1:81.00 ±16.99; T2:65.00 ±10.52; t(6) =-4.247; p-corrected=0.016) as well as across T0 and T2 (T0:77.00 ±15.34; T2: 65.00±10.52; t(6) =-3.340; p-corrected=0.047) in high-dependent participants.

### TOS and vitamin E

Considerable changes were found in both total oxidative stress (TOS) (χ^2^(2) =6.118; p=0.047) and levels of vitamin E (χ^2^(2) =13.104; p=0.001). Pairwise comparisons revealed that the levels of vitamin E were higher in T2 compared to those of T0 (W=-3.432; p-corrected=0.003).

Female participants showed a decreasing trend in TOS (χ^2^(2) =2.182; p=0.336) but significant increase in vitamin E levels (χ^2^(2) =7.302; p=0.026) across T0, T1, T2 evaluation phases. Changes in vitamin E levels in female participants, were mainly found between T0 and T2 (T0:14.60, [12.60, 16.10]; T2: 15.99, [15.58, 17.10]; W=-2.667; p=0.024). Similar outcomes were observed in male participants. TOS levels showed a decreasing trend across time (χ^2^(2) =5.333; p=0.069) whereas vitamin E levels were found to be increasing across time (χ^2^(2) =6.333; p=0.042).

Grouping participants based on Fageström score, TOS levels were significantly decreased across time (T0, T1, T2) in severe smokers (T0: 536.563, [444.416, 822.892]; T1: 493.713, [411.415, 863.515]; T2: 373.561, [291.629, 767.975]; χ^2^(2) =9.000; p=0.011) but not in moderate smokers, even though a decreasing trend was mentioned (T0: 621.696, [417.702, 971.400]; T1: 573.126, [339.990, 706.854]; T2: 533.500, [360.552, 799.560]; χ^2^(2) =1.273; p=0.529). Vitamin E levels were enhanced in both groups, both for moderate and severe smokers, across time (T0, T1, T2) (Moderate smokers: χ^2^(2) =8.512; p=0.014; Severe smokers: χ^2^(2) =6.333; p=0.042). Pairwise comparisons also showed that vitamin E levels were greater in moderate smokers in T2 compared to T0 (T0: 13.900, [10.500, 16.200]; T2:16.580, [15.640, 17.210]; W=-2.578; p-corrected=0.030). Although a similar trend was observed in severe smokers across T0 and T2, retained post-Bonferroni correction (T0: 14.605, [13.370, 14.930]; T2:16.010, [15.305, 17.210]; W=-2.201; p-corrected=0.084).

Splitting participants by pack-years showed that TOS levels declined only for high-dependent smokers across time conditions (T0, T1, T2) (T0: 524.556, [475.986, 971.400]; T1: 438.856, [414.842, 706.854]; T2: 360.552, [305.128, 417.702]; χ^2^(2) =11.143; p=0.004). Pairwise comparisons across time conditions indicated that the decrease in TOS levels was marginally achieved across T0 and T2 (W=-2.366; p-corrected=0.054) as well as across T1 and T2 (W=-2.366; p-corrected=0.054). On the other hand, vitamin levels increased across time only in low-dependent smokers (T0: 14.760, [10.545, 16.230]; T1: 15.490, [14.538, 16.543]; T2: 16.285, [15.460, 17.308]; χ^2^(2) =9.385; p=0.009). An increasing trend was displayed in high-dependent smokers (T0: 14.550, [12.680, 14.700]; T1: 16.420, [13.910, 17.800]; T2: 16.100, [15.580, 17.100]; χ^2^(2) =5.429; p=0.066). Comparing vitamin E levels across time in both groups (low/high-dependent smokers), showed a marginally significant increase was revealed across T0 and T2 (Low-dependent: W=-2.95; p=0.051; High-dependent: W=-2.366; p=0.054).

## Behavioral Data

### Self-administered questionnaires

Participants considerably decreased their degree of dependence across time as shown by the Fageström test of nicotine dependence [50] (T0: 5.00, [3.00, 8.00]; T1: 5.00, [1.50, 7.00]; T2: 4.00, [0.00, 6.50]; χ^2^(2) =7.042; p=0.030). Pairwise comparisons indicated that a significant decrease in the degree of nicotine dependence was achieved across T0 and T2 (W=-2.571; p=0.030; r= -0.624). Post-hoc tests did not reveal any significant change in the degree of dependence between genders (all p-values>0.05).

Motivation across time did not display any changes (χ^2^(2) =2.281; p=0.320). Post-hoc tests did not show any alteration in motivation levels across time (T0, T1, T2) depending on gender (Female: χ^2^(2) =1.805; p=0.406; Male: χ^2^(2) =2.696; p=0.260), intensity of dependence based on Fageström score (Moderate smokers: χ^2^(2) =0.326; p=0.850; Severe smokers: χ^2^(2) =4.667; p=0.097) and pack-years (Low-dependent: χ^2^(2) =2.513; p=0.285 ; High-dependent: χ^2^(2) =0.240; p=0.887).

The readiness to quit smoking appeared to be preserved across time (T0, T1, T2) (χ^2^(2) =2.800; p=0.247). Moreover, the Contemplation ladder score did not change in either female (χ^2^(2) =1.727; p=0.422) or male (χ^2^(2) =1.077; p=0.584) participants. Similar outcomes revealed a compared readiness to quit smoking across time based on Fageström score (Moderate smokers: χ^2^(2) =2.435; p=0.296; Severe smokers: χ^2^(2) =0.500; p=0.779) and pack-years (Low-dependent: χ^2^(2) =2.273; p=0.321; High-dependent: χ^2^(2) =2.923; p=0.232).

Withdrawal symptoms showed a decreasing trend across time (T0:26.53±11.170; T1: 24.24±10.287; T2: 22.65±10.167; F(2,32) =1.225; p=0.307). Post-hoc tests indicated that female and male participants did not show any change in the presence of withdrawal across time (Female: F(2,20) =2.533; p=0.105; Male: F(2,10) =0.069; p=0.934) even though a decreasing trend was present. Grouping participants depending on Fageström score, both moderate and severe smokers showed a decreasing trend in the withdrawal symptoms (Moderate smokers: F(2,20) =1.926; p=0.172; Severe smokers: F(2,10) =0.177; p=0.840). Similar outcomes revealed comparable withdrawal scores based on pack-years (Low-dependent: F(2,18) =1.056; p=0.368; High-dependent: F(2,12) =0.179; p=0.838).

Beck Depression Inventory (BDI) scores showed a downward trend across time (T0:14.00±9.394; T1: 13.35±9.347; T2: 12.06±8.511; F(2,32) =0.535; p=0.591). BDI scores seem to remain stable across time in both female (F(2,20) =0.410; p=0.669) and male participants (F(2,10) =0.220; p=0.807). A declining trend was found in BDI scores across time, dividing participants based on Fageström score (Moderate smokers: F(2,20) =0.225; p=0.801; Severe smokers: F(2,10)=1.390; p=0.293) and pack-years (Low-dependent: F(2,18) =0.436; p=0.653 ; High-dependent: F(2,12) =1.070; p=0.374).

A considerable decrease was found in the State Anxiety index across time (T0:45.06±13.288; T1: 39.18±10.376; T2: 36.71±12.066; F(2,32) =3.590; p=0.039). After grouping participants based on their gender, female (T0:42.00±12.767; T1: 37.45±9.554; T2: 35.00±12.830; F(1.290,12.896) =1.572; p=0.239) and male (T0:50.67±13.441; T1: 42.33±11.978; T2: 39.83±10.889; F(2,10) =1.956; p=0.192) participants showed a downward trend in State Anxiety index across time. Moderate smokers showed a marginal decrease in their State Anxiety scores across time (T0:46.36±12.808; T1: 38.45±11.264; T2: 36.09±13.464; F(2,20)=3.348; p=0.056) whereas a decreasing trend was exhibited in severe smokers (T0:42.67±15.042; T1: 40.50±9.354; T2: 37.83±10.048; F(2,10)=0.436; p=0.658).Dividing participants based on pack-years showed that low-dependent (T0:48.10±12.242; T1: 38.00±11.015; T2: 35.00±13.038; F(2,18)=4.841; p=0.021), but not high-dependent (T0:40.71±14.442; T1: 40.86±9.974; T2: 39.14±11.022; F(2,12) =0.107; p=0.899) had decreased State Anxiety scores across time.

By comparing Trait anxiety scores across time, a significant decline was observed (T0:47.71±11.329; T1: 43.71±10.913; T2: 41.24±11.940; F(2,32) =3.574; p=0.040). Pairwise comparisons showed that said decrease was achieved across T0 and T2 (t(16) =-3.180; p-corrected: 0.017). Post-hoc tests with respect to gender referred to a downward trend in Trait anxiety score across time in both female (F(2,20) =2.272; p=0.129) and male (F(2,10) =1.431; p=0.284) participants. Moreover, a decline in Trait anxiety scores across time was observed in moderate smokers (T0:49.91±11.545; T1: 42.64±13.208; T2: 41.55±9.637; F(2,20) =4.758; p=0.020) but not in severe (F(2,10) =0.762; p=0.492) smokers. Trait anxiety decrease was found across T0 and T2 (t(10) =-3.692; p-corrected=0.012) in moderate smokers.

Similar findings were observed when grouping participants with respect to pack-years. Low-dependent subjects showed decline in their Trait anxiety scores across time (T0: 50.00±11.832; T1: 42.60±13.015; T2: 40.00±10.770; F(2,18) =4.752; p=0.022) and particularly across T0 and T2 (t(9) =-3.932; p-corrected=0.010). High-dependent participants showed a declining trend (T0: 44.43±10.533; T1: 45.29±7.653; T2: 43.00±14.142; F(2,12) =0.274; p=0.765).

Self-esteem was found to be enhanced in participants across time (T0: 20.76±4.777; T1: 21.53±4.110; T2: 22.71±4.959; F(2,32) =3.374; p=0.047). Pairwise comparisons across time indicated that the increase was present across T0 and T2 but the change did not survive after multiple testing correction (t(16) =2.493; p-corrected=0.072). A marginal increase in self-esteem was observed in female participants (T0: 21.82±5.095; T1: 21.91±5.049; T2: 23.82±5.510; F(2,20) =3.291; p=0.058). Enhancement in self-esteem across time was documented in severe smokers (T0: 21.83±2.229; T1: 21.33±1.966; T2: 24.33±3.502; F(2,10) =5.602; p=0.023) but not in moderate smokers (F(2,20) =1.604; p=0.226). The increase in self-esteem of severe smokers was found across T0 and T2 (t(5) =3.727; p-corrected=0.041). Grouping subjects based on pack-years, both low and high-dependent participants showed an increasing trend (p-values >0.05).

Changes in general health test scores were not present across time (χ^2^(2) =3.633; p=0.163). Similar outcomes were observed in post-hoc tests performed based on gender (Female: χ^2^(2) =3.000; p=0.223; Male: χ2(2)=2.545; p=0.280), Fageström scores (Moderate smokers: χ^2^(2) =4.550; p=0.103 ; Severe smokers: χ^2^(2) =0.300; p=0.861) and pack-years (Low-dependent: χ^2^(2) =5.314; p=0.070; High-dependent: χ^2^(2) =0.560; p=0.756).

Quality of life indices were preserved across time in participants (Index: χ^2^(2) =2.042; p=0.360; Scale: χ^2^(2) =5.080; p=0.079). Post-hoc tests revealed that there were no changes in quality-of-life indices across time in female (Index: χ^2^(2) =3.500; p=0.174; Scale: χ^2^(2) =1.448; p=0.485) and male (Index: χ^2^(2) =0.001; p=1.000; Scale: χ^2^(2) =4.952; p=0.084) participants. Moderate (Index: χ^2^(2) =0.889; p=0.641; Scale: χ^2^(2) =1.750; p=0.417) and severe (Index: χ^2^(2) =3.500; p=0.174; Scale: χ^2^(2) =4.333; p=0.115) smokers did not show any alterations in quality-of-life parameters. Similar findings were revealed grouping participants based on pack-years (Low-dependent smokers - Index: χ^2^(2) =5.250; p=0.072; Scale: χ^2^(2) =3.500; p=0.174; High-dependent smokers - Index: χ^2^(2) =2.375; p=0.305; Scale: χ^2^(2) =3.909; p=0.142).

### Neuropsychological assessment

The completion time of Trail A task was preserved across time in participants (χ^2^(2) =4.750; p=0.093). Both Female (χ^2^(2) =2.176; p=0.337) and male (χ^2^(2) =3.273; p=0.195) participants showed no difference in their performance in Trail A across time. An enhancement of the Trail A task performance across time was observed in severe (χ^2^(2) =6.870; p=0.032) smokers but not in moderate (χ^2^(2) =0.424; p=0.809) smokers. The decrease in Trail A time was found across T0 and T2 (W=-2.207; p=0.027; p-corrected=0.081) but it did not survive Bonferroni correction. Likewise, grouping participants based on pack-years, showed that high-dependent participants exhibited greater performance in Trail A across time (χ^2^(2) =8.222; p=0.016). A marginal decrease in completion time of Trail A was found across T0 and T2 (W=-2.371; p=0.018; p-corrected=0.054). Low-dependent participants did not show any change in the completion time of Trail A task (χ^2^(2) =0.483; p=0.786). Participants appeared to retain the completion time of Trail B task across time (χ^2^(2) =1.661; p=0.436). Both female (χ^2^(2) =2.800; p=0.247) and male (χ^2^(2) =0.001; p=1.000) smokers did not show changes in the time needed for completing the Trail B task. Neither moderate smokers (χ^2^(2) =2.457; p=0.293) nor severe smokers (χ^2^(2) =3.000; p=0.223) showed changes in completion time of Trail B task. Similar outcomes were found after splitting participants based on pack-years (Low-dependent: χ^2^(2) =0.839; p=0.657; High-dependent: χ^2^(2) =3.429; p=0.180).

The participants’ performance on the Digit Span Forward task was preserved across time (χ^2^(2) =1.574; p=0.455). Changes in the Digit Span Forward task were absent in both female (χ^2^(2) =1.000; p=0.607) and male participants (χ^2^(2) =0.609; p=0.738). The performance of moderate (χ^2^(2) =3.920; p=0.141) and severe (χ^2^(2) =3.545; p=0.170) smokers respectively, did not change across time. Similar outcomes were found by grouping participants based on pack-years (Low-dependent: χ^2^(2) =3.545; p=0.170; High-dependent: χ^2^(2) =4.880; p=0.087).

Performance on the Digit Backward task did not show any alterations across time (χ^2^(2) =5.216; p=0.074). Digit Backward task scores changed across time in male (χ^2^(2) =6.778; p=0.034) but not for female (χ^2^(2) =3.152; p=0.207) participants. Although a decrease in the Digit Backward task score was found in males across T1 and T2, the change did not remain significant post-Bonferroni correction (T1:7.50, [5.75, 10.25]; T2:6.50, [5.50, 8.50]; W=-2.121; p=0.034; p-corrected=0.102).

The performance of moderate (χ^2^(2) =4.647; p=0.098) and severe (χ^2^(2) =1.412; p=0.494) participants in the Digit Backward task did not change across time. Similar outcomes were found grouping participants based on pack-years (Low-dependent: χ^2^(2) =2.867; p=0.239; High-dependent: χ^2^(2) =2.667; p=0.264).

Participants did not modify their Digit Span total scores across time (χ^2^(2) =2.778; p=0.249). Both female (χ^2^(2) =2.067; p=0.356) and male (χ^2^(2) =1.333; p=0.513) participants preserved their performance on Digit Span across time. Digit Span total scores changed in severe (χ^2^(2) =7.043; p=0.030) smokers but not in moderate (χ^2^(2) =1.355; p=0.508) smokers. Pairwise comparisons revealed an increase across T0 and T1 (T0: 13.50, [12.00, 16.25]; T1: 16.00, [13.75, 18.25]; W=-2.032; p=0.042) which was not present after Bonferroni correction. A marginal change in Digit Span total scores was revealed in high-dependent (χ^2^(2) =5.769; p=0.056) but not in low-dependent participants (χ^2^(2) =0.001; p=1.000). Pairwise comparisons showed an increase across T0 and T2 (T0: 14.00, [12.00, 17.00]; T2: 16.00, [14.00, 20.00]; W=-2.251; p=0.024; p-corrected= 0.072) which was not present after Bonferroni correction.

Participants did not display any change in their performance on the Stroop words task across time (χ^2^(2) =2.370; p=0.306). Female smokers changed their Stroop words scores across time (T0: 118.00, [109.50, 121.50]; T1: 107.00, [102.50, 121.00], T2: 122.00, [112.50, 125.00]; χ^2^(2) =6.059; p=0.048) whereas similar findings were not observed in male participants (χ^2^(2) =0.700; p=0.705). An improvement in the Stroop words task was revealed in female participants across T1 and T2 (T1: 117.00, [105.00, 122.00], T2: 123.00, [113.75, 126.25]; χ^2^(2) =-2.552; p=0.033). Stroop words scores did not change across time when grouping subjects based on Fageström scores (Moderate (χ^2^(2) =2.529; p=0.282); Severe (χ^2^(2) =0.300; p=0.861) and pack-years (Low-dependent: (χ^2^(2) =0.069; p=0.966); High-dependent: (χ^2^(2) =3.920; p=0.141).

Stroop colors task scores seem to be preserved across time in participants (χ^2^(2) =2.714; p=0.257). Meanwhile, female (χ^2^(2) =1.647; p=0.439) and male (χ^2^(2) =1.455; p=0.483) smokers did not display any change in their performance on the Stroop word task across time. Similar findings were observed grouping participants with respect to Fageström scores (Moderate: χ^2^(2) =0.182; p=0.913); Severe: χ^2^(2) =4.261; p=0.119) and pack-years (Low-dependent: (χ^2^(2) =0.483; p=0.786); High-dependent: (χ^2^(2) =3.630; p=0.163).

Total Stroop words-colors scores increased across time (T0, T1, T2) (χ^2^(2) =14.982; p=0.001). More specifically, participants improved their total Stroop scores across T0 and T2 (T0: 51.50, [46.25, 57.25], T2: 60.00, [50.25, 70.75]; χ^2^(2) =-3.301; p=0.003). Significant improvement in total Stroop words-colors scores was documented in female (χ^2^(2) =9.879; p=0.007) participants but not in male subjects (χ^2^(2) =5.182; p=0.075). Female participants increased their scores across T0 and T2 (T0: 53.00, [49.75, 60.00], T2: 67.00, [56.25, 72.00]; W=-2.670; p-corrected=0.024). Additionally, moderate, but not severe smokers (χ^2^(2) =5.727; p=0.057), changed total Stroop scores across time (χ^2^(2) =9.515; p=0.009). Moderate smokers, in particular, improved their performance on Stroop tests across T0 and T2 (T0: 53.00, [48.00, 65.25], T2: 64.00, [55.75, 75.00]; W= -2.668; p-corrected= 0.024). Changes in total Stroop scores across time were present for both low-dependent (χ^2^(2) =6.867; p=0.032) and high-dependent (χ^2^(2) =8.240; p=0.016) smokers. Comparing scores across time conditions in pairs, significant improvement was displayed only for low-dependent subjects across T0 and T2 (T0: 52.00, [47.00, 65.50], T2: 61.00, [54.50, 75.00]; W= -2.527; p-corrected= 0.036). A similar finding was observed in high-dependent subjects, but it did not persist after multiple testing corrections (T0: 50.00, [46.00, 55.00], T2: 51.00, [46.00, 70.00]; W= -2.207; p=0.027; p-corrected= 0.081).

## Electrophysiological Data

### Biofeedback

#### Across baseline

No considerable alterations in baseline temperature were observed across sessions (χ 2(4) =4.000; p=0.406). Temperature in baseline measurements across sessions appeared to remain stable in both female (χ 2(4) =3.273; p=0.513) and male participants (χ 2(4) =2.800; p=0.592). Similar outcomes in baseline temperature across sessions were observed when grouping participants based on their Fageström score (Moderate (χ^2^(4) =4.727; p=0.316); Severe (χ^2^(4) =2.533; p=0.639) and pack-years (Low-dependent: (χ^2^(4) =2.800; p=0.592); High-dependent: (χ^2^(2) =2.629; p=0.622).

#### Baseline vs. Corresponding session

Moreover, a greater temperature was observed in most sessions, except for session 1, compared to the corresponding baseline (session 2 vs. baseline 2: W=-2.627; p=0.009; session 3 vs. baseline 3: W=-2.817; p=0.005; session 4 vs. baseline 4: W=-2.675; p= 0.007; session 5 vs. baseline 5: W=-3.051; p=0.002).

Female participants showed increased temperature in sessions 4 and 5 compared to the corresponding baseline measurements (session 4 vs. baseline 4: W=-1.956; p= 0.050; session 5 vs. baseline 5: W=-2.401; p=0.016). Male participants displayed a significant increase in their temperature in sessions 2 (session 2 vs. baseline 2: W=-1.992; p=0.046), 3 (session 3 vs. baseline 3: W=-2.201; p=0.028) and 5 (session 5 vs. baseline 5: W=-2.203; p=0.043), when compared to corresponding baseline temperature.

An increase in Temperature was observed during all sessions, apart from session 1, compared to the corresponding baseline measurement in moderate smokers (session 2 vs. baseline 2: W=-2.490; p=0.013; session 3 vs. baseline 3: W=-1.956; p=0.050; session 4 vs. baseline 4: W=-2.578; p= 0.010; session 5 vs. baseline 5: W=-2.803; p=0.005). For severe smokers, temperature measurements during session 3 were found to have increased, compared to the corresponding baseline (session 3 vs. baseline 3: W=-2.201; p=0.028).

Grouping participants based on pack-years, low-dependent smokers showed increased temperature during sessions 2 (session 2 vs. baseline 2: W=-2.191; p=0.028), 4 (session 4 vs. baseline 4: W=-2.599; p=0.009) and 5 (session 5 vs. baseline 5: W=-2.666; p=0.008) compared to the corresponding baseline measurement. On the other hand, high-dependent smokers increased their hand temperature during session 3, when compared to the resting-measurement (session 3 vs. baseline 3: W=-2.366; p=0.018).

#### Across sessions

There were no observed changes in temperature across sessions (χ 2(4) =6.588; p=0.159). An increasing trend was documented in sessions 4 and 5 compared to session 1 (session 4 vs. session 1: W=-2.320; p=0.020; p-corrected=0.180; session 5 vs. session 1: W=-2.249; p=0.025; p-corrected=0.225). Female (χ 2(4) =4.873; p=0.301) and male (χ^2^(4) =3.867; p=0.424) participants did not show any alterations in hand temperature across sessions. The temperature across sessions seems to be preserved in both moderate and severe smokers (Moderate: χ^2^(4) =6.982; p=0.137; Severe: χ^2^(4) =5.333; p=0.255). Similar findings were observed when splitting participants based on pack-years (Low-dependent smokers: χ^2^(4) =7.360; p=0.118; High-dependent smokers: χ^2^(4) =1.829; p=0.767).

### Neurofeedback

#### Across baseline

Changes in theta and alpha amplitude across all sessions baselines were not observed (Theta: χ^2^(19) =13.460; p=0.814; Alpha: χ 2(19) =21.609; p=0.304). Marginally significant changes were found in the baseline theta/alpha ratio across sessions (χ 2(19) =29.917; p=0.053).

Modifications in baseline amplitude of the theta and alpha bands as well as theta/alpha ratio were not observed in female (Theta: χ^2^(19) =10.495; p=0.940; Alpha: χ^2^(19) =17.361; p=0.565; Theta/alpha ratio: χ 2(19)=24.255; p=0.187) and male (Theta: χ^2^(19) =22.470; p=0.261; Alpha: χ^2^(19) =16.476; p=0.625; Theta/alpha ratio: χ^2^(19) =11.841; p=0.892) participants.

Baseline measurements in theta and alpha amplitude did not show any changes across sessions for either moderate (Theta: χ^2^(19) =14.492; p=0.754; Alpha: χ^2^(19) =16.175; p=0.646) or severe (Theta: χ^2^(19) =15.343; p=0.701; Alpha: χ^2^(19) =24.326; p=0.184) smokers. Significant alterations in theta/alpha ratio were observed for moderate (χ^2^(19) =31.142; p=0.039) but not for severe smokers (χ^2^(19) =15.233; p=0.708).

The amplitude of the theta and alpha bands did not display any changes in baseline measurements for neither low nor high dependent smokers (Low-dependent – Theta: χ^2^(19) =11.202; p=0.917; Alpha: χ^2^(19) = 12.208; p=0.877; High-dependent Theta: χ^2^(19) = 17.243; p=0.573; Alpha: χ^2^(19) =23.275; p=0.225). Theta/alpha ratios seem to be modified in low-dependent (χ^2^(19) =32.604; p=0.027) but not for high-dependent (χ^2^(19) =14.292; p=0.766) smokers.

#### Baseline vs. Corresponding session

Participants displayed an increased theta amplitude during the 8th session (Baseline: 8.879, [6.790, 14.069]; Session:12.405, [7.590, 15.097]; W=-1.965; p=0.049), 15th session (Baseline: 9.835, [6.506, 14.465]; Session: 10.479, [6.766, 15.575]; W=-3.290; p=0.001) and 17th session (Baseline: 8.131, [6.488, 13.946]; Session: 9.116, [7.259, 16.589]; W=-2.059; p=0.039), compared to the corresponding baseline.

The amplitude of the alpha band decreased in 2nd (Baseline: 11.788, [7.068, 27.192]; Session: 9.981, [7.879, 18.186]; W=-2.059; p=0.039), 4th (Baseline: 14.541, [8.282, 26.392]; Session: 10.348, [7.764, 19.937]; W=-2.012; p=0.044), 8th (Baseline: 12.162, [8.198, 27.192]; Session: 11.224, [7.200, 20.013]; W=-2.438; p=0.015), 11th (Baseline: 13.641, [8.065, 25.444]; Session: 11.640, [6.918, 20.484]; W=-2.296; p=0.022), 12th (Baseline: 11.368, [7.949, 32.397]; Session: 10.661, [7.686, 21.127]; W=-2.012; p=0.044), 19th (Baseline: 12.633, [8.284, 24.601]; Session: 10.988, [6.948, 23.305]; W=-2.012; p=0.044) and 20th (Baseline: 12.119, [8.243, 27.430]; Session: 10.882, [7.192, 22.898]; W=-2.201; p=0.028) session compared to the corresponding baseline measurement. An increase in alpha amplitude, relative to the corresponding baseline, was observed in the 10th (Baseline: 10.545, [8.211, 29.119]; Session: 11.640, [7.466, 18.796]; W=-2.959; p=0.003) and 13th (Baseline: 10.926, [8.172, 28.589]; Session: 12.231, [6.904, 18.105]; W=-2.201; p=0.028) sessions. The theta/alpha ratio was increased for the majority of sessions, with the first and second one being the only exceptions, compared to the baseline.

**Table S1**. Theta/alpha ratio eyes-closed resting-state measurements before training and during session, test for statistical significance and p-values, for participants, by session.

|  | **Theta/alpha ratio pre vs. corresponding session, EC resting-state measurement** | | |
| --- | --- | --- | --- |
| Session | Before training (baseline/pre) | Session | Test statistic, p-value |
| 1 | 0.949, [0.779, 1.540] | 1.276, [0.989, 1.634] | W=-1,775; p=0.076 |
| 2 | 0.986, [0.759, 1.316] | 1.249, [0.882, 1.495] | W=-1,917; p=0.055 |
| 3 | 0.966, [0.626, 1.188] | 1.134, [0.986, 1.447] | W=-3,101; p=0.002 |
| 4 | 0.868, [0.682, 1.245] | 1.216, [0.907, 1.562] | W=-3,479; p=0.001 |
| 5 | 0.910, [0.638, 1.181] | 1.204, [0.886, 1.579] | W=-2,911; p=0.004 |
| 6 | 0.938, [0.626, 1.219] | 1.140, [0.762, 1.589] | W=-2,580; p=0.010 |
| 7 | 0.843, [0.713, 1.235] | 1.158, [0.801, 1.535] | W=-2,580; p=0.010 |
| 8 | 0.866, [0.611, 1.104] | 1.196, [0.953, 1.524] | W=-3,621; p<0.001 |
| 9 | 0.851, [0.727, 1.174] | 1.276, [0.768, 1.518] | W=-3,290; p=0.001 |
| 10 | 0.896, [0.673, 1.118] | 1.249, [1.008, 1.412] | W=-3,621; p<0.001 |
| 11 | 0.934, [0.720, 1.072] | 1.151, [0.926, 1.478] | W=-3,195; p=0.001 |
| 12 | 0.898, [0.626, 1.076] | 1.168, [0.898, 1.361] | W=-3,621; p<0.001 |
| 13 | 0.886, [0.617, 1.211] | 1.220, [0.996, 1.557] | W=-3,385; p=0.001 |
| 14 | 0.928, [0.684, 1.171] | 1.190, [0.836, 1.474] | W=-2,864; p=0.004 |
| 15 | 0.875, [0.653, 1.268] | 1.264, [0.873, 1.565] | W=-2,722; p=0.006 |
| 16 | 0.877, [0.738, 1.117] | 1.170, [0.866, 1.506] | W=-3,527; p<0.001 |
| 17 | 0.806, [0.655, 1.134] | 1.247, [0.868, 1.460] | W=-3,385; p=0.001 |
| 18 | 0.878, [0.640, 1.127] | 1.276, [0.898, 1.440] | W=-3,148; p=0.002 |
| 19 | 0.931, [0.631, 1.134] | 1.236, [0.902, 1.646] | W=-3,053; p=0.002 |
| 20 | 0.890, [0.675, 1.172] | 1.159, [0.793, 1.562] | W=-3,337; p=0.001 |

Female participants showed an increased theta/alpha ratio in all sessions apart from session 1 and session 7, compared to the corresponding baseline. On the other side, male participants showed increments in theta/alpha ratio in sessions 4, 7, 8, 9, 10, 12 compared to the corresponding baseline measurements.

**Table S2**. Theta/alpha ratio eyes-closed resting-state measurements before training and during session, test for statistical significance and p-values, for female participants, by session.

| **Female participants** | **Theta/alpha ratio pre vs. corresponding session, EC resting-state measurement** | | |
| --- | --- | --- | --- |
| Session | Before training (baseline/pre) | Session | Test statistic, p-value |
| 1 | 1.004, [0.784, 1.570] | 1.314, [1.093, 1.673] | W=-1.511; p=0.131 |
| 2 | 0.986, [0.765, 1.216] | 1.325, [1.029, 1.370] | W=-2.134; p=0.033 |
| 3 | 0.968, [0.628, 1.141] | 1.134, [1.077, 1.412] | W=-2.667; p=0.008 |
| 4 | 0.868, [0.705, 1.233] | 1.231, [0.954, 1.564] | W=-2.667; p=0.008 |
| 5 | 0.910, [0.680, 1.194] | 1.204, [0.992, 1.528] | W=-2.49; p=0.013 |
| 6 | 1.004, [0.860, 1.142] | 1.162, [0.871, 1.563] | W=-2.401; p=0.016 |
| 7 | 0.843, [0.729, 1.219] | 1.189, [0.917, 1.358] | W=-1.867; p=0.062 |
| 8 | 0.926, [0.659, 1.081] | 1.196, [1.090, 1.392] | W=-2.934; p=0.003 |
| 9 | 0.890, [0.761, 1.156] | 1.276, [0.874, 1.461] | W=-2.667; p=0.008 |
| 10 | 0.896, [0.706, 1.070] | 1.249, [1.041, 1.368] | W=-2.934; p=0.003 |
| 11 | 0.940, [0.791, 1.073] | 1.151, [0.933, 1.445] | W=-2.845; p=0.004 |
| 12 | 0.905, [0.657, 1.050] | 1.168, [0.914, 1.267] | W=-2.934; p=0.003 |
| 13 | 0.994, [0.637, 1.172] | 1.281, [1.139, 1.553] | W=-2.934; p=0.003 |
| 14 | 0.949, [0.688, 1.188] | 1.190, [0.885, 1.445] | W=-2.312; p=0.021 |
| 15 | 0.875, [0.713, 1.176] | 1.264, [1.117, 1.525] | W=-2.312; p=0.021 |
| 16 | 0.887, [0.819, 1.104] | 1.218, [1.061, 1.491] | W=-2.934; p=0.003 |
| 17 | 0.806, [0.657, 1.070] | 1.247, [1.024, 1.379] | W=-2.845; p=0.004 |
| 18 | 0.904, [0.643, 1.063] | 1.276, [0.966, 1.419] | W=-2.667; p=0.008 |
| 19 | 0.938, [0.735, 1.075] | 1.236, [0.981, 1.447] | W=-2.667; p=0.008 |
| 20 | 0.904, [0.707, 1.149] | 1.276, [0.970, 1.447] | W=-2.845; p=0.004 |

**Table S3**. Theta/alpha ratio eyes-closed resting-state measurements before training and during session, test for statistical significance and p-values, for male participants, by session.

| **Male participants** | **Theta/alpha ratio pre vs. corresponding session, EC resting-state measurement** | | |
| --- | --- | --- | --- |
| Session | Before training (baseline/pre) | Session | Test statistic, p-value |
| 1 | 0.855, [0.590, 1.567] | 1.127, [0.565, 1.658] | W=-0.943; p=0.345 |
| 2 | 1.081, [0.522, 2.627] | 0.974, [0.514, 1.728] | W=-0.524; p=0.600 |
| 3 | 0.836, [0.416, 1.546] | 1.127, [0.492, 1.606] | W=-1.363; p=0.173 |
| 4 | 0.826, [0.476, 1.496] | 1.077, [0.492, 1.672] | W=-2.201; p=0.028 |
| 5 | 0.826, [0.483, 1.302] | 1.023, [0.491, 1.713] | W=-1.572; p=0.116 |
| 6 | 0.792, [0.505, 1.804] | 0.987, [0.481, 1.747] | W=-1.153; p=0.249 |
| 7 | 0.827, [0.471, 1.546] | 0.871, [0.678, 1.812] | W=-1.992; p=0.046 |
| 8 | 0.784, [0.502, 1.521] | 1.060, [0.683, 1.976] | W=-2.201; p=0.028 |
| 9 | 0.781, [0.478, 1.526] | 1.054, [0.511, 1.866] | W=-2.201; p=0.028 |
| 10 | 0.800, [0.459, 1.538] | 1.177, [0.831, 1.724] | W=-2.201; p=0.028 |
| 11 | 0.815, [0.503, 1.267] | 1.245, [0.671, 1.612] | W=-1.572; p=0.116 |
| 12 | 0.768, [0.495, 1.453] | 1.106, [0.615, 1.735] | W=-2.201; p=0.028 |
| 13 | 0.756, [0.503, 1.630] | 1.097, [0.632, 1.776] | W=-1.572; p=0.116 |
| 14 | 0.824, [0.498, 1.217] | 1.158, [0.475, 1.800] | W=-1.572; p=0.116 |
| 15 | 0.915, [0.498, 1.702] | 1.138, [0.639, 1.699] | W=-1.572; p=0.116 |
| 16 | 0.807, [0.468, 1.560] | 0.901, [0.562, 1.763] | W=-1.782; p=0.075 |
| 17 | 0.772, [0.471, 1.539] | 1.168, [0.571, 1.827] | W=-1.782; p=0.075 |
| 18 | 0.727, [0.453, 1.580] | 1.151, [0.748, 1.931] | W=-1.572; p=0.116 |
| 19 | 0.774, [0.447, 1.657] | 1.131, [0.561, 1.885] | W=-1.363; p=0.173 |
| 20 | 0.729, [0.534, 1.581] | 0.923, [0.684, 1.825] | W=-1.572; p=0.116 |

Moderate smokers enhanced their theta/alpha ratio in 16 out of 20 sessions compared to the corresponding resting-measurement, whereas for severe smokers it was displayed for 12 out of 20 sessions.

**Table S4**. Theta/alpha ratio eyes-closed resting-state measurements before training and during session, test for statistical significance and p-values, for moderate smoker participants, by session.

| **Moderate participants** | **Theta/alpha ratio pre vs. corresponding session, EC resting-state measurement** | | |
| --- | --- | --- | --- |
| Session | Before training (baseline/pre) | Session | Test statistic, p-value |
| 1 | 1.004, [ 0.775, 1.570] | 1.276, [1.007, 1.681] | W=-1.245; p=0.213 |
| 2 | 0.986, [0.765, 1.402] | 1.249, [0.963, 1.620] | W=-1.245; p=0.213 |
| 3 | 0.981, [0.628, 1.235] | 1.134, [1.060, 1.633] | W=-2.49; p=0.013 |
| 4 | 0.785, [0.705, 1.258] | 1.216, [0.954, 1.570] | W=-2.667; p=0.008 |
| 5 | 0.839, [0.607, 1.168] | 1.201, [0.939, 1.6229] | W=-2.312; p=0.021 |
| 6 | 0.908, [0.646, 1.296] | 1.051, [0.834, 1.563] | W=-2.045; p=0.041 |
| 7 | 0.843, [0.729, 1.252] | 1.189, [0.805, 1.712] | W=-1.6; p=0.110 |
| 8 | 0.866, [0.659, 1.081] | 1.196, [1.048, 1.392] | W=-2.934; p=0.003 |
| 9 | 0.890, [0.715, 1.192] | 1.201, [0.721, 1.574] | W=-2.49; p=0.013 |
| 10 | 0.877, [0.666, 1.070] | 1.249, [1.036, 1.450] | W=-2.934; p=0.003 |
| 11 | 0.934, [0.699, 0.990] | 1.061, [0.933, 1.348] | W=-2.667; p=0.008 |
| 12 | 0.905, [0.614, 1.103] | 1.115, [0.914, 1.267] | W=-2.934; p=0.003 |
| 13 | 0.886, [0.625, 1.251] | 1.194, [1.017, 1.553] | W=-2.756; p=0.006 |
| 14 | 0.827, [0.688, 1.124] | 1.161, [0.885, 1.445] | W=-2.045; p=0.041 |
| 15 | 0.875, [0.695, 1.360] | 1.264, [0.979, 1.605] | W=-1.867; p=0.062 |
| 16 | 0.877, [0.727, 1.086] | 1.170, [0.734, 1.520] | W=-2.756; p=0.006 |
| 17 | 0.790, [0.657, 1.199] | 1.182, [0.830, 1.414] | W=-2.845; p=0.004 |
| 18 | 0.878, [0.643, 1.191] | 1.159, [0.885, 1.419] | W=-2.401; p=0.016 |
| 19 | 0.938, [0.734, 1.194] | 1.162, [0.825, 1.873] | W=-2.578; p=0.010 |
| 20 | 0.890, [0.675, 1.194] | 1.159, [0.800, 1.676] | W=-2.845; p=0.004 |

**Table S5**. Theta/alpha ratio eyes-closed resting-state measurements before training and during session, test for statistical significance and p-values, for severe smoker participants, by session.

| **Severe participants** | **Theta/alpha ratio pre vs. corresponding session, EC resting-state measurement** | | |
| --- | --- | --- | --- |
| Session | Before training (baseline/pre) | Session | Test statistic, p-value |
| 1 | 0.877, [0.743, 1.422] | 1.299, [0.755, 1.614] | W= -1.153; p=0.249 |
| 2 | 0.985, [0.596, 1.331] | 1.235, [0.665, 1.450] | W=-1.782; p=0.075 |
| 3 | 0.917, [0.573, 1.129] | 1.267, [0.811, 1.436] | W=-1.782; p=0.075 |
| 4 | 0.914, [0.586, 1.272] | 1.155, [0.758, 1.560] | W=-2.201; p=0.028 |
| 5 | 0.946, [0.614, 1.339] | 1.224, [0.749, 1.354] | W=-1.782; p=0.075 |
| 6 | 0.988, [0.569, 1.295] | 1.284, [0.639, 1.618] | W=-1.572; p=0.116 |
| 7 | 0.798, [0.641, 1.188] | 1.060, [0.724, 1.462] | W=-2.201; p=0.028 |
| 8 | 0.922, [0.552, 1.341] | 1.229, [0.674, 1.721] | W=-2.201; p=0.028 |
| 9 | 0.844, [0.678, 1.087] | 1.331, [0.737, 1.511] | W=-2.201; p=0.028 |
| 10 | 0.915, [0.645, 1.243] | 1.255, [0.708, 1.422] | W=-2.201; p=0.028 |
| 11 | 1.006, [0.671, 1.456] | 1.548, [0.799, 1.612] | W=-1.782; p=0.075 |
| 12 | 0.894, [0.620, 1.155] | 1.226, [0.816, 1.488] | W=-2.201; p=0.028 |
| 13 | 0.991, [0.577, 1.338] | 1.327, [0.734, 1.604] | W=-1.992; p=0.046 |
| 14 | 1.041, [0.631, 1.265] | 1.365, [0.690, 1.553] | W=-1.992; p=0.046 |
| 15 | 0.977, [0.567, 1.285] | 1.331, [0.705, 1.547] | W=-1.992; p=0.046 |
| 16 | 0.984, [0.737, 1.266] | 1.148, [0.896, 1.551] | W=-2.201; p=0.028 |
| 17 | 0.815, [0.596, 1.262] | 1.264, [0.829, 1.566] | W=-1.992; p=0.046 |
| 18 | 0.857, [0.565, 1.286] | 1.333, [0.937, 1.571] | W=-1.992; p=0.046 |
| 19 | 0.859, [0.507, 1.292] | 1.302, [0.884, 1.539] | W=-1.782; p=0.075 |
| 20 | 0.843, [0.644, 1.258] | 1.160, [0.726, 1.448] | W=-1.572; p=0.116 |

When grouping participants based on pack-years, low-dependent individuals showed increments in their theta/alpha ratio compared to the baseline in 14 out of 20 sessions, while high-dependent smokers in 18 out 20 sessions.

**Table S6**. Theta/alpha ratio eyes-closed resting-state measurements before training and during session, test for statistical significance and p-values, for low-dependent participants, by session.

| **Low-dependent participants** | **Theta/alpha ratio pre vs. corresponding session, EC resting-state measurement** | | |
| --- | --- | --- | --- |
| Session | Before training (baseline/pre) | Session | Test statistic, p-value |
| 1 | 0.976, [0.736, 1.577] | 1.269, [0.875, 1.611] | W=-0.255; p=0.799 |
| 2 | 0.931, [0.708, 1.413] | 1.199, [0.730, 1.419] | W=-0.561; p=0.575 |
| 3 | 0.974, [0.576, 1.165] | 1.109, [0.750, 1.441] | W=-2.293; p=0.022 |
| 4 | 0.749, [0.649, 1.170] | 1.146, [0.769, 1.335] | W=-2.497; p=0.013 |
| 5 | 0.778, [0.582, 1.061] | 1.096, [0.727, 1.554] | W=-1.988; p=0.047 |
| 6 | 0.884, [0.615, 1.170] | 1.044, [0.746, 1.457] | W=-1.682; p=0.093 |
| 7 | 0.831, [0.665, 1.227] | 1.056, [0.768, 1.431] | W=-0.866; p=0.386 |
| 8 | 0.843, [0.624, 1.010] | 1.169, [0.823, 1.374] | W=-2.803; p=0.005 |
| 9 | 0.871, [0.661, 1.165] | 1.185, [0.669, 1.490] | W=-2.497; p=0.013 |
| 10 | 0.831, [0.573, 1.044] | 1.103, [0.968, 1.389] | W=-2.803; p=0.005 |
| 11 | 0.878, [0.653, 0.971] | 1.057, [0.870, 1.254] | W=-2.497; p=0.013 |
| 12 | 0.807, [0.588, 1.063] | 1.022, [0.827, 1.265] | W=-2.803; p=0.005 |
| 13 | 0.844, [0.596, 1.156] | 1.173, [0.902, 1.409] | W=-2.599; p=0.009 |
| 14 | 0.764, [0.622, 1.038] | 1.148, [0.784, 1.302] | W=-1.784; p=0.074 |
| 15 | 0.839, [0.646, 1.169] | 1.239 ,[0.743, 1.484] | W=-1.682; p=0.093 |
| 16 | 0.848, [0.663, 0.962] | 1.165, [0.690, 1.431] | W=-2.599; p=0.009 |
| 17 | 0.755, [0.609, 1.047] | 1.152, [0.773, 1.388] | W=-2.701; p=0.007 |
| 18 | 0.850, [0.596, 1.090] | 1.147, [0.857, 1.357] | W=-2.191; p=0.028 |
| 19 | 0.934, [0.662, 1.105] | 1.160, [0.767, 1.554] | W=-2.395; p=0.017 |
| 20 | 0.836, [0.644, 1.161] | 1.144, [0.782, 1.504] | W=-2.497; p=0.013 |

**Table S7**. Theta/alpha ratio eyes-closed resting-state measurements before training and during session, test for statistical significance and p-values, for high-dependent participants, by session.

| **High-dependent participants** | **Theta/alpha ratio pre vs. corresponding session, EC resting-state measurement** | | |
| --- | --- | --- | --- |
| Session | Before training (baseline/pre) | Session | Test statistic, p-value |
| 1 | 0.886, [0.784, 1.510] | 1.351, [1.093, 1.673] | W=-2.028; p=0.043 |
| 2 | 1.210, [0.759, 1.402] | 1.325, [1.029, 1.687] | W=-2.366; p=0.018 |
| 3 | 0.930, [0.625, 1.522] | 1.412, [1.077, 1.470] | W=-2.197; p=0.028 |
| 4 | 0.938, [0.659, 1.390] | 1.231, [0.954, 1.564] | W=-2.366; p=0.018 |
| 5 | 0.982, [0.680, 1.217] | 1.244, [1.037, 1.632] | W=-2.028; p=0.043 |
| 6 | 1.037, [0.606, 1.756] | 1.427, [0.691, 1.624] | W=-1.859; p=0.063 |
| 7 | 0.864, [0.697, 1.523] | 1.158, [0.917, 1.773] | W=-2.366; p=0.018 |
| 8 | 1.021, [0.563, 1.429] | 1.262, [1.048, 1.920] | W=-2.366; p=0.018 |
| 9 | 0.848, [0.739, 1.502] | 1.386, [0.815, 1.764] | W=-2.197; p=0.028 |
| 10 | 0.934, [0.706, 1.477] | 1.261, [1.249, 1.567] | W=-2.366; p=0.018 |
| 11 | 1.071, [0.740, 1.324] | 1.511, [0.933, 1.606] | W=-2.028; p=0.043 |
| 12 | 0.898, [0.657, 1.435] | 1.284, [1.025, 1.642] | W=-2.366; p=0.018 |
| 13 | 1.096, [0.609, 1.562] | 1.433, [1.017, 1.733] | W=-2.197; p=0.028 |
| 14 | 1.124, [0.827, 1.188] | 1.381, [0.787, 1.700] | W=-2.197; p=0.028 |
| 15 | 1.135, [0.612, 1.614] | 1.509, [1.117, 1.612] | W=-1.859; p=0.063 |
| 16 | 1.104, [0.825, 1.522] | 1.218, [0.998, 1.733] | W=-2.366; p=0.018 |
| 17 | 0.824, [0.657, 1.439] | 1.280, [0.906, 1.746] | W=-2.028; p=0.043 |
| 18 | 0.904, [0.637, 1.454] | 1.390, [0.966, 1.905] | W=-2.197; p=0.028 |
| 19 | 0.904, [0.527, 1.516] | 1.329, [0.981, 1.844] | W=-2.028; p=0.043 |
| 20 | 0.904, [0.674, 1.516] | 1.276, [0.786, 1.763] | W=-2.197; p=0.028 |

#### Across sessions

Theta amplitude seems to be preserved for all sessions (χ^2^(19) =18.511; p=0.489). Across sessions, theta amplitude did not display any segregation between participants based on their gender (Female: χ^2^(19) =19.393; p=0.432; Male: χ^2^(19) =25.029; p=0.160) or degree of nicotine dependence (Moderate: χ^2^(19) =21.065; p=0.333; Severe: χ^2^(19) =20.874; p=0.344; Low-dependent: χ^2^(19) =25.775; p=0.137; High-dependent: χ^2^(19) =20.365; p=0.373).

Alterations in alpha amplitude were not observed across sessions (χ^2^(19) =14.396; p=0.760). Alpha amplitude, however, seemed to be preserved across sessions in both female (χ^2^(19) =19.393; p=0.432) and male (χ^2^(19) =16.909; p=0.596) participants. Similar outcomes were revealed after grouping participants with respect to the degree of nicotine dependence (Moderate: χ^2^(19) =23.065; p=0.235; Severe: χ^2^(19) =25.932; p=0.132; Low-dependent: χ^2^(19) =18.071; p=0.518; High-dependent: χ^2^(19) =21.436; p=0.313).

Modifications of the theta/alpha ratio were not observed across sessions (χ^2^(19) =14.462; p=0.756). Similar findings were observed when grouping participants with respect to their gender (Female: χ^2^(19) =17.850; p=0.532; Male: χ^2^(19) =19.324; p=0.436), Fageström scores (Moderate: χ^2^(19) =19.755; p=0.409; Severe: χ^2^(19) =23.072; p=0.234) and pack-years (Low-dependent: χ^2^(19) =18.840; p=0.467; High-dependent: χ^2^(19) =8.792; p=0.977).

#### Pre-post sessions

Participants showed an enhanced theta amplitude during the 6th (Baseline: 8.772, [7.404, 14.038];Post-training:10.695, [7.655, 14.400]; W=-2.485; p=0.013), 15th (Baseline: 9.835, [6.506, 14.465];Post-training :12.700, [6.433, 17.763]; W=-2.769; p=0.006) and 20th (Baseline: 9.435, [7.020, 14.837];Post-training :10.206, [7.248, 18.924]; W=-2.343; p=0.019) post-training measurements, compared to the corresponding baseline.

Theta amplitude was increased during the 6th (Baseline: 9.072, [7.305, 13.619]; Post-training: 11.901, [9.764, 13.929]; W=-2.045; p=0.041) and 15th (Baseline: 11.813, [6.523, 14.275]; Post-training: 13.002, [6,444, 18.544]; W=-2.401; p=0.016) session, for female participants. Male participants showed an increased theta amplitude during the 8th (Baseline: 8.233, [5.396, 15.236]; Post-training:9.194 [7.227, 16.689]; W=-2.201; p= 0.028), 12th (Baseline: 8.581, [5.922, 15.026]; Post-training: 9.683, [7.354, 17.393 ]; W=-1.992; p=0.046) and 17th (Baseline: 7.356, [5.040, 13.911]; Post-training:8.576, [6.277, 17.104]; W=-1.992; p=0.046) sessions, when comparing post-training resting-state with baseline measurement.

Moderate smokers showed higher theta amplitude post-training compared to the corresponding baseline in 7 out of 20 sessions (Baseline 6: 9.959, [7.606,14.458]; Post-training 6: 13.116, [10.187, 14.872]; W=-2.134; p=0.033; Baseline 11: 11.991, [7.979, 14.137]; Post-training 11: 14.023, [9.133, 16.892]; W=-2.134; p= 0.033; Baseline 15:11.974, [6.489, 14.654]; Post-training 15: 13.634, [6.423, 18.544]; W=-2.045; p=0.041; Baseline 16: 12.209, [7.356,15.930]; Post-training 16: 14.087, [7.853, 18.575]; W=-2.045; p=0.041; Baseline 17:12.024, [7.041,14.016]; Post-training 17: 13.102, [9.352, 16.592]; W=-2.401; p=0.016; Baseline 19: 12.856, [7.380, 14.484]; Post-training 19: 13.624, [8.360, 17.353]; W=-2.490; p=0.013; Baseline 20: 12.367, [7.380, 15.228]; Post-training 20: 15.166, [8.360, 20.764]; W=-2.134; p=0.033). We did not observe any changes in theta amplitude for severe smokers (all p-values>0.050).

Likewise, low-dependent smokers showed increments in theta amplitude after training in 5 out of 20 sessions (Baseline 6: 9.516, [7.989, 13.853]; Post-training 6: 13.140 , [10.046, 16.016]; W=-2.497; p=0.013; Baseline 15: 11.894, [6.514, 15.099]; Post-training: 13.318, [9.708, 17.384], W=-2.191; p=0.028; Baseline 17: 11.677, [7.295, 13.911]; Post-training 17: 12.726, [9.792, 17.166]; W=-2.497; p=0.013; Baseline 19: 12.243, [7.768, 14.585]; Post-training: 13.681, [8.542, 17.527]; W=-2.395; p=0.017; Baseline 20: 11.678, [7.857, 16.325]; Post-training 20: 13.882, [8.528, 21.231]; W=-1.988; p=0.047) compared to baseline whereas no changes were reported in high-dependent smokers (all p-values>0.050).

Comparing alpha amplitude before and after NF training (at each session), showed a decreasing trend (all-p-values>0.05) in the majority of sessions apart from the 12th and 13th. A decrease in alpha amplitude was found in 2 out of 20 sessions (Baseline 8: 12.162, [8.724, 31.277]; Post-training 8: 12.155, [8.772, 25.807]; W=-2.312; p=0.021; Baseline 9: 14.499, [9.920, 22.752]; Post-training 9: 11.017, [7.758, 14.408], W=-2.223; p=0.026) in female participants comparing changes in amplitude before and after training. Changes in alpha amplitude were not observed in male participants (all p-values>0.050). A decrease in alpha amplitude after training was indicated in 8th session (Baseline 8: 17.986, [7.671, 31.277]; Post-training 8: 13.091, [7.574, 25.807]; W=-2.223; p=0.026) for moderate smokers as well as in 11th session (Baseline 11: 9.769, [6.039, 25.237]; Post-training 11: 9.410, [5.713, 21.870]; W=-2.201; p=0.028) in severe smokers. Additionally, we did not observe any alterations in alpha amplitude after grouping subjects based on pack-years (all p-values>0.050). The trained band (theta/alpha ratio) was showed to be enhanced compared to the corresponding baseline in all sessions apart from the first three sessions and 7th session (p>0.05).

**Table S8.** Theta/alpha ratio eyes-closed resting-state measurements before training and after training, test for statistical significance and p-values, for all participants, by session.

|  | **Theta/alpha ratio post vs. pre, EC resting-state measurement** | | |
| --- | --- | --- | --- |
| Session | Before training (baseline/pre) | After training (post) | Test statistic, p-value |
| 1 | 0.949, [0.779, 1.540] | 1.227, [0.845, 1.425] | W= -0.024; p=0.981 |
| 2 | 0.986, [0.759, 1.316] | 1.064, [0.763, 1.442] | W= -0.118; p=0.906 |
| 3 | 0.966, [0.626, 1.188] | 1.041, [0.834, 1.345] | W= -1.917; p=0.055 |
| 4 | 0.868, [0.682, 1.245] | 1.061, [0.879, 1.373] | W= -2.438; p=0.015 |
| 5 | 0.910, [0.638, 1.181] | 1.092, [0.830, 1.345] | W= -2.107; p=0.035 |
| 6 | 0.938, [0.626, 1.219] | 1.423, [0.779, 1.628] | W= -2.817; p=0.005 |
| 7 | 0.843, [0.713, 1.235] | 0.979, [0.701, 1.446] | W= -0.497; p=0.619 |
| 8 | 0.866, [0.611, 1.104] | 1.137, [0.723, 1.399] | W= -2.769; p=0.006 |
| 9 | 0.851, [0.727, 1.174] | 1.172, [0.746, 1.646] | W= -2.627; p=0.009 |
| 10 | 0.896, [0.673, 1.118] | 1.097, [0.695, 1.480] | W= -2.675; p=0.007 |
| 11 | 0.934, [0.720, 1.072] | 1.208, [0.905, 1.531] | W= -3.053; p=0.002 |
| 12 | 0.898, [0.626, 1.076] | 0.990, {0.744, 1.461] | W= -2.485; p=0.013 |
| 13 | 0.886, [0.617, 1.211] | 1.312, [0.744, 1.594] | W= -2.580; p=0.010 |
| 14 | 0.928, [0.684, 1.171] | 1.176, [0.878, 1.497] | W= -2.343; p=0.019 |
| 15 | 0.875, [0.653, 1.268] | 1.455, [0.781, 1.645] | W= -2.059; p=0.039 |
| 16 | 0.877, [0.738, 1.117] | 1.113, [0.787, 1.300] | W = -2.107; p= 0.035 |
| 17 | 0.806, [0.655, 1.134] | 1.058, [0.738, 1.528] | W= -2.391; p= 0.017 |
| 18 | 0.878, [0.640, 1.127] | 1.165, [1.039, 1.596] | W=-3.053; p=0.002 |
| 19 | 0.931, [0.631, 1.134] | 1.298, [0.861, 1.571] | W=-2,722; p=0.006 |
| 20 | 0.890, [0.675, 1.172] | 0.909. [0.695, 1.600] | W=2,059; p= 0.039 |

Female participants had an increase in theta/alpha ratio after training, relative to the corresponding baseline, in 12 out of 20 sessions whereas similar findings were observed in 1 out of 20 sessions in male participants (Baseline 12: 0.768, [0.495, 1.453]; Post-training 12: 1.082, [0.615, 1.832]; W=-1.992; p=0.046) (see [1]).

**Table S9.** Theta/alpha ratio eyes-closed resting-state measurements before training and after training, test for statistical significance and p-values, for female participants, by session.

| **Female participants** | **Theta/alpha ratio post vs. pre EC resting-state measurement** | | |
| --- | --- | --- | --- |
| Session | Before training (baseline/pre) | After training (post) | Test statistic, p-value |
| 1 | 1.004, [0.784, 1.570] | 1.227, [0.909, 1.352] | W=-0.178; p=0.859 |
| 2 | 0.986, [0.765, 1.216] | 1.064, [1.008, 1.508] | W=-1.067; p=0.286 |
| 3 | 0.968, [0.628, 1.141] | 1.041, [0.982, 1.388] | W=-2.312; p=0.021 |
| 4 | 0.868, [0.705, 1.233] | 1.061, [0.965, 1.477] | W=-2.49; p=0.013 |
| 5 | 0.910, [0.680, 1.194] | 1.092, [0.909, 1.293] | W=-1.778; p=0.075 |
| 6 | 1.004, [0.860, 1.142] | 1.423, [0.865, 1.633] | W=-2.578; p=0.010 |
| 7 | 0.843, [0.729, 1.219] | 1.072, [0.824, 1.445] | W=-0.089; p=0.929 |
| 8 | 0.926, [0.659, 1.081] | 1.137, [0.753, 1.274] | W=-2.312; p=0.021 |
| 9 | 0.890, [0.761, 1.156] | 1.172, [0.898, 1.715] | W=-2.49; p=0.013 |
| 10 | 0.896, [0.706, 1.070] | 1.202, [0.852, 1.397] | W=-2.312; p=0.021 |
| 11 | 0.940, [0.791, 1.073] | 1.238, [0.926, 1.581] | W=-2.845; p=0.004 |
| 12 | 0.905, [0.657, 1.050] | 0.990, [0.846, 1.222] | W=-1.6; p=0.110 |
| 13 | 0.994, [0.637, 1.172] | 1.465, [0.904, 1.573] | W=-2.49; p=0.013 |
| 14 | 0.949, [0.688, 1.188] | 1.210, [0.899, 1.558] | W=-1.956; p=0.050 |
| 15 | 0.875, [0.713, 1.176] | 1.455, [0.855, 1.531] | W=-1.6; p=0.110 |
| 16 | 0.887, [0.819, 1.104] | 1.113, [0.901, 1.337] | W=-1.778; p=0.075 |
| 17 | 0.806, [0.657, 1.070] | 1.058, [0.931, 1.471] | W=-1.956; p=0.050 |
| 18 | 0.904, [0.643, 1.063] | 1.211, [1.060, 1.586] | W=-2.49; p=0.013 |
| 19 | 0.938, [0.735, 1.075] | 1.298, [1.019, 1.567] | W=-2.667; p=0.008 |
| 20 | 0.904, [0.707, 1.149] | 1.019, [0.697, 1.547] | W=-1.423; p=0.155 |

Moderate smokers displayed an increased theta/alpha ratio in 11 out of 20 sessions, whereas for severe smokers, the theta/alpha ratio was increased in 2 out of 20 sessions (Baseline 4: 0.914, [0.586, 1.272]; Post-training 4:1.105, [0.638, 1.559]; W=-2.201; p=0.028; Baseline 18: 0.857, [0.565, 1.286] ; Post-training 18: 1.145, [1.081, 1.664]; W=1.992; p=0.046).

**Table S10**. Theta/alpha ratio eyes-closed resting-state measurements before training and after training, test for statistical significance and p-values, for moderate smoker participants, by session.

| **Moderate participants** | **Theta/alpha ratio post vs. pre EC resting-state measurement** | | |
| --- | --- | --- | --- |
| Session | Before training (baseline/pre) | After training (post) | Test statistic, p-value |
| 1 | 1.004, [0.775, 1.570] | 1.227, [0.901, 1.384] | W=-0.800; p=0.424 |
| 2 | 0.986, [0.765, 1.402] | 1.274, [1.008, 1.534] | W=-0.533; p=0.594 |
| 3 | 0.981, [0.628, 1.235] | 1.239, [0.982, 1.388] | W=-1.778; p=0.075 |
| 4 | 0.785, [0.705, 1.258] | 1.053, [0.938, 1.269] | W=-1.511; p=0.131 |
| 5 | 0.839, [0.607, 1.168] | 1.171, [0.868, 1.396] | W=-2.045; p=0.041 |
| 6 | 0.908, [0.646, 1.296] | 1.423, [0.823, 1.795] | W=-2.578; p=0.010 |
| 7 | 0.843, [0.729, 1.252] | 0.878, [0.674, 1.448] | W=-0.622; p=0.534 |
| 8 | 0.866, [0.659, 1.081] | 1.137, [0.815, 1.361] | W=-2.934; p=0.003 |
| 9 | 0.890, [0.715, 1.192] | 1.108, [0.717, 1.661] | W=-1.778; p=0.075 |
| 10 | 0.877, [0.666, 1.070] | 1.297, [0.688, 1.563] | W=-2.756; p=0.006 |
| 11 | 0.934, [0.699, 0.990] | 1.197, [0.926, 1.481] | W=-2.756; p=0.006 |
| 12 | 0.905, [0.614, 1.103] | 0.875, [0.803, 1.443] | W=-1.956; p=0.050 |
| 13 | 0.886, [0.625, 1.251] | 1.465, [0.842, 1.615] | W=-2.223; p=0.026 |
| 14 | 0.827, [0.688, 1.124] | 1.090, [0.899, 1.621] | W=-2.134; p=0.033 |
| 15 | 0.875, [0.695, 1.360] | 1.455, [0.855, 1.531] | W=-1.334; p=0.182 |
| 16 | 0.877, [0.727, 1.086] | 0.981, [0.784, 1.426] | W=-2.045; p=0.041 |
| 17 | 0.790, [0.657, 1.199] | 1.019, [0.650, 1.584] | W=-1.689; p=0.091 |
| 18 | 0.878, [0.643, 1.191] | 1.211, [1.019, 1.605] | W=-2.401; p=0.016 |
| 19 | 0.938, [0.734, 1.194] | 1.298, [0.835, 1.575] | W=-2.312; p=0.021 |
| 20 | 0.890, [0.675, 1.194] | 0.909, [0.694, 1.652] | W=-1.689; p=0.091 |

Likewise, low-dependent smokers had an increased theta/alpha ratio in 8 out of 20 sessions. Improvement in theta/alpha ratio was observed in 2 out of 20 sessions in high-dependent smokers (Baseline 18: 0.904, [0.637, 1.454]; Post-training 18:1.211, [1.138, 1.895]; W=-2.197; p=0.028; Baseline 20: 0.904, [0.674, 1.516]; Post-training 20: 1.138, [0.747, 1.751]; W=-2.197; p=0.028).

**Table S11**. Theta/alpha ratio eyes-closed resting-state measurements before training and after training, test for statistical significance and p-values, for low dependent participants, by session.

| **Low-dependent participants** | **Theta/alpha ratio post vs. pre EC resting-state measurement** | | |
| --- | --- | --- | --- |
| Session | Before training (baseline/pre) | After training (post) | Test statistic, p-value |
| 1 | 0.976, [0.736, 1577] | 0.950, [0.720, 1.384] | W=-1.478; p=0.139 |
| 2 | 0.931, [0.708, 1.413] | 1.036, [0.675, 1.651] | W=-0.561; p=0.575 |
| 3 | 0.974, [0.576, 1.165] | 1.166, [0.655, 1.459] | W=-1.988; p=0.047 |
| 4 | 0.749, [0.649, 1.170] | 0.997, [0.745, 1.320] | W=-2.191; p=0.028 |
| 5 | 0.778, [0.582, 1.061] | 0.955, [0.692, 1.319] | W=-1.682; p=0.093 |
| 6 | 0.884, [0.615, 1.170] | 1.179, [0.752, 1.673] | W=-1.988; p=0.047 |
| 7 | 0.831, [0.665, 1.227] | 0.860, [0.635, 1.251] | W=-1.172; p=0.241 |
| 8 | 0.843, [0.624, 1.010] | 1.082, [0.746, 1.260] | W=-2.701; p=0.007 |
| 9 | 0.871, [0.661, 1.165] | 1.030, [0.660, 1.675] | W=-2.09; p=0.037 |
| 10 | 0.831, [0.573, 1.044] | 1.027, [0.574, 1.438] | W=-2.395; p=0.017 |
| 11 | 0.878, [0.653, 0.971] | 1.151, [0.778, 1.506] | W=-2.599; p=0.009 |
| 12 | 0.807, [0.588, 1.063] | 0.873, [0.675, 1.278] | W=-1.58; p=0.114 |
| 13 | 0.844, [0.596, 1.156] | 1.184, [0.620, 1.573] | W=-1.886; p=0.059 |
| 14 | 0.764, [0.622, 1.038] | 1.027, [0.771, 1.357] | W=-1.58; p=0.114 |
| 15 | 0.839, [0.646, 1.169] | 1.342, [0.780, 1.491] | W=-1.58; p=0.114 |
| 16 | 0.848, [0.663, 0.962] | 0.941, [0.617, 1.359] | W=-1.58; p=0.114 |
| 17 | 0.755, [0.609, 1.047] | 0.975, [0.619, 1.499] | W=-1.58; p=0.114 |
| 18 | 0.850, [0.596, 1.090] | 1.158, [0.980, 1.563] | W=-2.191; p=0.114 |
| 19 | 0.934, [0.662, 1.105] | 1.158, [0.730, 1.569] | W=-1.886; p=0.059 |
| 20 | 0.836, [0.644, 1.161] | 0.873, [0.608, 1.573] | W=-1.172; p=0.241 |

### EEG

#### Graph properties

Possible alterations in different graph properties of PLI networks at the theta, alpha and beta bands were explored. An increasing trend was observed in the clustering coefficient (CC) and global efficiency (EF) of theta PLI networks across time (CC – T0: 0.151, [0.148, 0.153]; T1: 0.152, [0.150, 0.155]; T2: 0.155, [0.150, 0.158]; χ^2^(2) =5.059; p=0.080; EF – T0: 0.283, [0.277, 0.285]; T1: 0.285, [0.279, 0.288]; T2: 0.288, [0.279, 0.291]; χ^2^(2) =1.529; p=0.465). A decreasing trend was shown in the characteristic path length (CPL) of theta PLI networks across time (CPL – T0: 3.772, [3.738, 3.858]; T1: 3.745, [3.723, 3.801]; T2: 3.708, [3.680, 3.800]; χ^2^(2) =1.529; p=0.465). Alterations in the alpha PLI network properties across time were not present even though a downward trend was shown in the characteristic path length (CPL – T0: 3.767, [3.654, 3.795]; T1: 3.757, [3.667, 3.822]; T2: 3.727, [3.684, 3.855]; χ^2^(2) =0.824; p=0.662). Furthermore, we did not observe any modifications in graph properties of beta PLI networks across time (all p-values>0.05).

# Acknowledgement

Icon images used in figures and tables were freely downloaded from the website: https://www.flaticon.com/
